# Integrative systems biology of wheat susceptibility to *Fusarium graminearum* uncovers a conserved gene regulatory network and identifies master regulators targeted by fungal core effectors

**DOI:** 10.1186/s12915-024-01852-x

**Published:** 2024-03-05

**Authors:** Florian Rocher, Samir Dou, Géraldine Philippe, Marie-Laure Martin, Philippe Label, Thierry Langin, Ludovic Bonhomme

**Affiliations:** 1 UMR 1095 Génétique Diversité Ecophysiologie Des Céréales, Université Clermont Auvergne, INRAE, Clermont-Ferrand, France; 2 Université Paris-Saclay, CNRS, INRAE, Université Evry, Institute of Plant Sciences Paris-Saclay (IPS2), Gif Sur Yvette, 91190 France; 3 Université de Paris, Institute of Plant Sciences Paris-Saclay (IPS2), Gif Sur Yvette, 91190 France; 4 UMR MIA Paris-Saclay, AgroParisTech, INRAE, Université Paris-Saclay, Gif Sur Yvette, France; 5 Physique Et Physiologie Intégratives de L’Arbre en Environnement Fluctuant, Université Clermont Auvergne, INRAE, UMR 547, Aubière, Cedex France

**Keywords:** *Triticum aestivum*, *Fusarium graminearum*, System biology approach, Regulation network, Susceptibility factors

## Abstract

**Background:**

Plant diseases are driven by an intricate set of defense mechanisms counterbalanced by the expression of host susceptibility factors promoted through the action of pathogen effectors. In spite of their central role in the establishment of the pathology, the primary components of plant susceptibility are still poorly understood and challenging to trace especially in plant-fungal interactions such as in Fusarium head blight (FHB) of bread wheat. Designing a system-level transcriptomics approach, we leveraged the analysis of wheat responses from a susceptible cultivar facing *Fusarium graminearum* strains of different aggressiveness and examined their constancy in four other wheat cultivars also developing FHB.

**Results:**

In this study, we describe unexpected differential expression of a conserved set of transcription factors and an original subset of master regulators were evidenced using a regulation network approach. The dual-integration with the expression data of pathogen effector genes combined with database mining, demonstrated robust connections with the plant molecular regulators and identified relevant candidate genes involved in plant susceptibility, mostly able to suppress plant defense mechanisms. Furthermore, taking advantage of wheat cultivars of contrasting susceptibility levels, a refined list of 142 conserved susceptibility gene candidates was proposed to be necessary host’s determinants for the establishment of a compatible interaction.

**Conclusions:**

Our findings emphasized major FHB determinants potentially controlling a set of conserved responses associated with susceptibility in bread wheat. They provide new clues for improving FHB control in wheat and also could conceivably leverage further original researches dealing with a broader spectrum of plant pathogens.

**Supplementary Information:**

The online version contains supplementary material available at 10.1186/s12915-024-01852-x.

## Background

The ascomycete fungus *Fusarium graminearum* (teleomorph *Gibberella zeae*) is the main causal agent of the Fusarium head blight (FHB) disease on bread wheat [1, 2]. Causing significant yield losses, grain quality alterations, and the accumulation of carcinogenic mycotoxins (e.g., deoxynivalenol, DON) [3–5], FHB represents a major threat for wheat production [6, 7] and results in critical economic losses reaching up to USD 1.176 billion over 2015 and 2016 in the USA [8]. With rising temperatures and occasional increases in air humidity due to climate change, FHB outbreaks are expected to be more frequent and severe [9, 10], making it necessary to develop efficient and sustainable management strategies [11]. Wheat resistance to FHB is a complex and strictly quantitative trait with more than 625 reported quantitative trait loci (QTLs) that displayed relatively minor effects especially when environmental conditions are conducive to the pathogen [12, 13]. Hence, further researches are needed to identify alternative sources of resistance to improve breeding strategies and to efficiently control FHB.

For the last 20 years, the multiple evidences of plant genes required for pathogen infection [14] have opened new opportunities to control natural immunity in plants. These genes, the so-called susceptibility genes, are involved in a wide range of plant fundamental processes that are hijacked by the pathogen through the delivery of a large repertoire of effectors [15–18]. Resistance based on the mutation of susceptibility genes (S genes) represents a promising alternative in breeding resistant cultivars, as exemplify by the broad-spectrum resistance to powdery mildew conferred by the natural mutation of the Mlo gene in barley [19]. In the wheat—*F. graminearum* pathosystem—the role of S genes in the interaction outcome has already been demonstrated in several studies. For instance, the deletion of specific chromosome fragments led to an increase of the resistance level, indicating the existence of key FHB S genes in the wheat genome [20–23]. Recently, direct functional evidences of the importance of FHB susceptibility factors in wheat were brought and revealed a high application value in increasing wheat resistance to *F. graminearum* [24–30], the most striking example being the natural mutation in the TaHRC gene that might underlie the resistance conferred by the Fhb1 QTL [27, 28]. Thus, identify the FHB S genes in wheat that drive the compatibility of the interaction with *F. graminearum* turns out to be a powerful way to expand the sources of sustainable resistance.

Mining a reliable catalogue of S genes requires to deepen our understanding of the molecular crosstalk between the two protagonists of the interaction [31, 32]. Biotic stress responses are based on complex signaling pathways involving signal perception, signal transduction, and expression of stress-responsive genes [33]. Through their role in triggering gene expression, transcription factors (TFs) play a pivotal role in the signal transduction step and orchestrate coordinated plant responses. As a consequence, TFs represent hub nodes of the stress molecular responses enabling the activation or the repression of a large set of downstream target genes [33–35]. Although their characteristics make them important pathogen’s targets to control host plant responses [18, 36] and thus interesting gene candidates to take advantage of complex trait improvement, the complexity of their regulation network requires to identify the downstream genes involved in the stress responses as well as all the impacted biological processes. Many studies have already reported the nature and diversity of wheat responses to *F. graminearum* using successful large-scale -omics studies [37–48]. However, no attempt has been made to decipher the complexity of the underlying regulation networks and its robustness when fungal strain of different aggressiveness and cultivars of contrasted susceptibilities are considered.

**Fig. 1:**
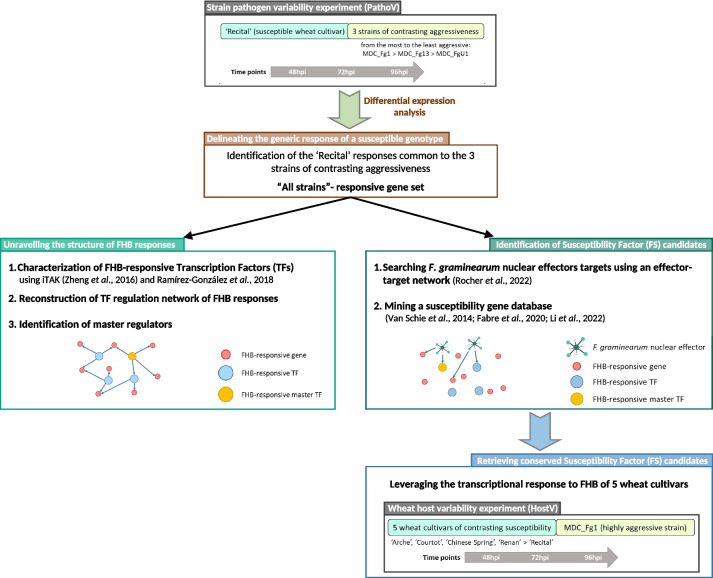
Overview of the approach used to decipher the mechanisms underlying wheat susceptibility to FHB and to identify relevant susceptibility genes

In this study, the molecular components underlying FHB susceptibility in bread wheat as well as their regulation processes are addressed using a mRNA sequencing (RNAseq)-based profiling of responsive genes during a time course FHB infection. This comprehensive analysis has been conducted by merging information from (i) the characterization of the regulation network orchestrating the responses of a highly susceptible wheat cultivar facing three *F. graminearum* strains of contrasting aggressiveness, (ii) the identification of S gene candidates through the integration of wheat responses to FHB with the expression data of fungal effector genes [49], and (iii) the characterization of the expression of these S gene candidates in different wheat cultivars of contrasting susceptibility (Fig. 1).

## Results

### FHB development in bread wheat cultivars

**Fig. 2:**
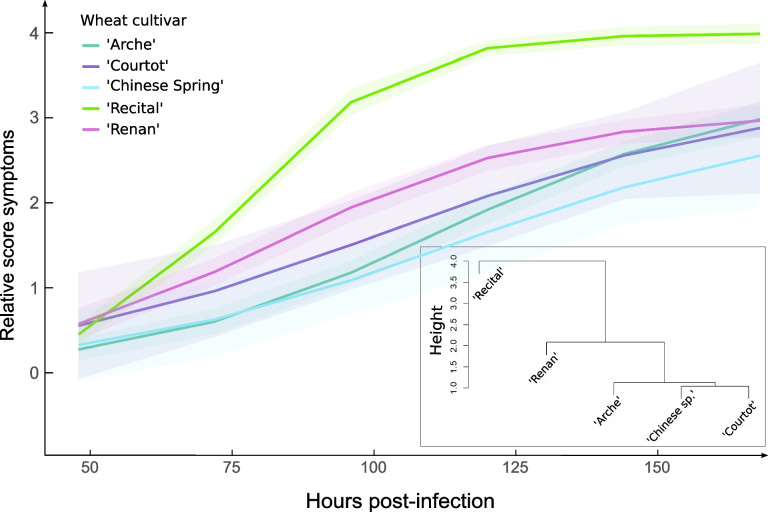
Dynamics of symptom development in the cultivars “Arche,” “Courtot,” “Chinese Spring,” “Recital,” and “Renan.” For each cultivar, symptom development was evaluated according to a logistic model (colored curves). The inset clustering tree represents the HAC performed on the logistic parameters

The dynamics of symptom development was evaluated in the five wheat cultivars using a logistic model applied on the measured score symptoms. Although no significant difference was observed for the maximum score symptom (*Asym*) reached at 168 h post inoculation (hpi), the time required to reach 50% of this maximum score (*xmid*) was significantly shorter in “Recital” than in “Arche,” “Courtot,” and “Chinese Spring” (Fig. 2). “Renan” also reached 50% of the maximum score symptom (*xmid*) in a significantly shorter time than both “Arche” and “Chinese Spring.” In “Recital,” the time required to switch from 50 to 75% of the maximum score symptom (*scal*) was significantly lower than the one measured in other cultivars except in “Renan.” To refine the susceptibility ranking of the five cultivars, we performed a hierarchical ascending clustering (HAC) on the three logistic regression parameters (Fig. 2 inset). It clearly discriminated “Recital” from the four other cultivars that were divided into two distinct groups with “Renan” that was separated from “Arche,” “Chinese Spring,” and “Courtot.” Based on the disease progress modeling, a susceptibility ranking of the cultivars was established as follows: “Recital” (high susceptibility), “Renan” (intermediate susceptibility), “Arche,” “Courtot,” and “Chinese Spring” (moderate susceptibility).

### Transcriptional landscape of early FHB responses in the most susceptible wheat genotype “Recital” facing different fungal strains revealed conserved changes

**Fig. 3:**
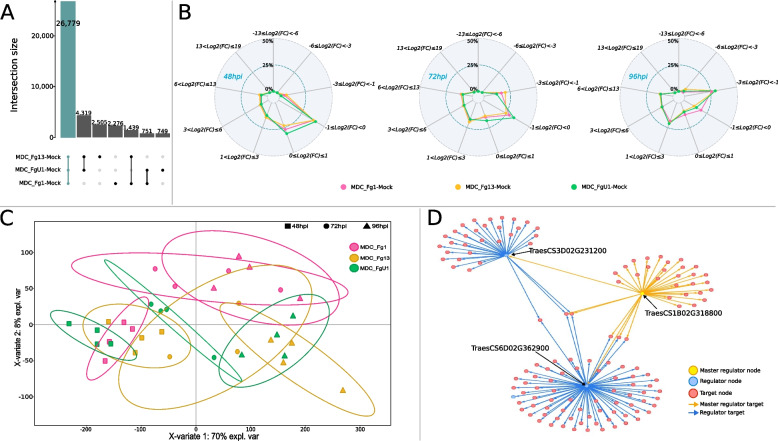
Characterization of “Recital” responses to the three strains of contrasting aggressiveness. **A** The structure of the FHB-responsive gene set is represented by an upset plot describing the number of genes significant for one (“Strain specific”-responsive gene set), two (“Strain accessory”-responsive gene set), or three (“All Strain”-responsive gene set, blue bar) strain-control comparisons. **B** The variation amplitudes of gene expression for each strain-control comparison at 48 hpi, 72 hpi, and 96 hpi were represented with spider charts. Spider chart describes the log2(FC) between each strain (MDC_Fg1 in pink, MDC_Fg13 in yellow and MDC_FgU1 in green) and control. Colored points indicate the proportion of genes belonging to each of the nine log2(FC) ranges. Extreme values were the maximum and minimum log2(FC) of all time points and strain control comparisons. A negative log2(FC) indicates a lower expression level in the infected condition than in the control one, and a positive log2(FC) indicates a higher expression level in infected condition than in the control one. Spider plots are composed of two grid circles, the middle grid circle indicating 25% and the maximum grid circle indicating 50%. **C** “Recital” experimental conditions were classified according to the “All strain”-responsive gene set. PLS-DA method was applied on the 26,779 genes responsive to the three *F. graminearum* strains to predict the strain-infection progress combinations. The plots of the individuals extracted from the PLS-DA are represented on the two first components. For each condition, confidence ellipses are plotted to highlight discrimination strength (level set to 95%). **D** The complexity of the regulation network controlling “Recital” FHB responses is illustrated by a directional network plot. Network was built using TF genes as regulators of all FHB responsive genes, meaning that some TF genes are targets of other TFs, the master regulators. The figure shows the targets of one master regulator (yellow node), TraesCS1B02G318800. Direct edges connecting the master regulator to its targets are indicated by yellow arrow. Master regulator’s target genes are indicated in blue for the TF genes and in red for the not TF genes. Blue arrows represent the direct edges between the regulated TF genes and their own targets

A total of 49,505 genes were found to be expressed in Recital and were used for the differential expression (DE) analysis. DE results were classified into 16 categories (Additional file 1: Table S1). Among the 41,891 differentially expressed genes (DEGs), we selected genes differentially expressed in response to at least one fungal strain in comparison to the control (M) conditions (treatment and interaction effects). This resulted in a FHB-responsive gene set composed of 38,818 genes (92.6% of the whole DE gene set, Fig. 3A) including 26,779 genes that were systematically regulated whatever the inoculated fungal strain (“All strains”-responsive gene set; 69% of the FHB-responsive gene set). Only 6509 and 5530 genes were differentially expressed in response to two of the three strains (“Strain accessory”-responsive gene set) and to one strain (“Strain specific”-responsive gene set), respectively. Overall, the magnitude of “Recital” responses to the three strains increased along with the infection progress (Fig. 3B). At 48 hpi, around 66% of the differences in gene expression between MDC_Fg1-, MDC_Fg13-, MDC_FgU1-inoculated and mock plants (fold changes, FC) did not exceed a twofold increase or decrease (− 1 ≤ *Log2*(FC) ≤ 1). At 72 hpi and 96 hpi in contrast, FC greater than twofold increase or decrease (*Log2*(FC) <  − 1 or *Log2*(FC) > 1) accounted for an average of 56% and 76% of the FHB-responsive genes whatever the inoculated strain, respectively.

To gain a better understanding of the strain impacts on wheat gene expression along with the infection progress, we computed a partial least square discriminant analysis (PLS-DA) on the “All strains”-responsive gene set trying to discriminate strain × time-point combinations (Fig. 3C). The first component, explaining 70% of the total variance, clearly discriminated the three infection stages, with 72 hpi acting as a transition phase between 48 and 96 hpi. The second component, explaining 8% of the variance, slightly discriminated the wheat responses to MDC_Fg1 from the ones induced with the two other strains at 72 hpi and 96 hpi. These results were confirmed by the HAC of the infected samples on the “All strains”-responsive gene set (Additional file 2: Fig. S1). In line with this, 93% of the “All strains”-responsive genes did not display any strain effect, emphasizing that “Recital” cultivar displayed similar quantitative responses to the three strains.

### The FHB development induced major readjustments in the abundance of key wheat transcriptional regulators independently of strain aggressiveness

**Fig. 4:**
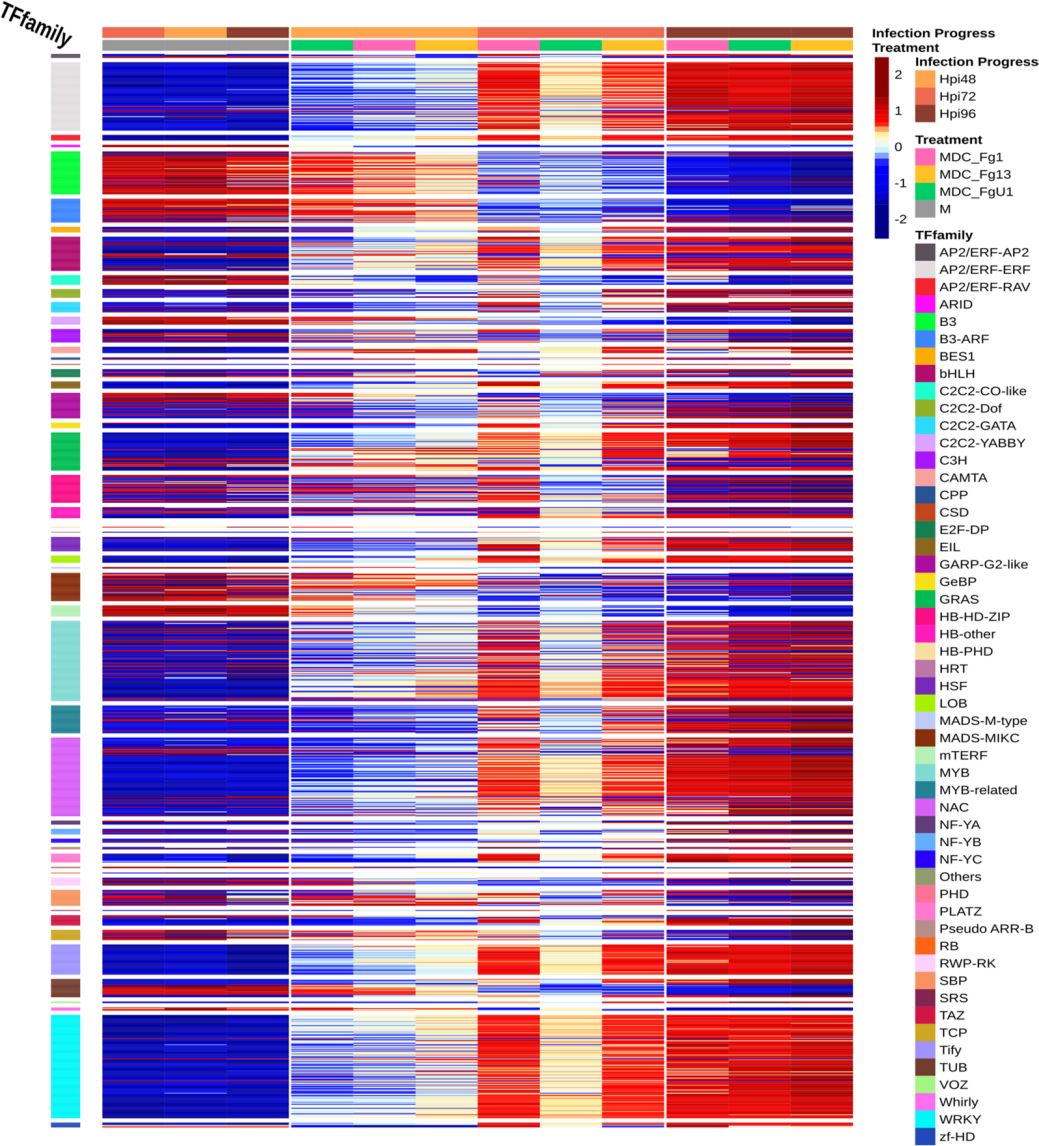
Expression regulation patterns of the “All Strain”-responsive gene set responsive TF encoding genes along with the infection progress “Recital.” The structure of gene and sample data sets were determined by HAC based on Ward’s minimum variance method using the z-score transformed gene expression values. Heatmap color scales represent the *z*-score transformed expression values of the TF genes from the All strain-responsive gene set for each condition. The clustering on top of the heatmap represents the experimental conditions which are labeled according to the factors infection progress and treatment. On the right side of the heatmap, TF genes were grouped and colored according to their TF family

The “All strains”-responsive genes demonstrated a reprogramming and temporal adjustments of the “Recital” transcriptome along the infection progress with two balanced gene groups (Additional file 2: Fig. S1). The first group, gathering 12,160 genes, displayed higher expression levels in control samples than in the infected ones, with a continuously decreasing gene expression along with the infection progress. On the opposite, the second group, gathering 14,619 genes, displayed lower expression levels in the control samples than in the infected ones depicting a continuously increasing gene expression along with the infection progress. The search of transcription factors among this “All strains”-responsive gene set identified 1074 TF encoding genes belonging to 53 TF families. In comparison with the whole expressed gene set, the “All strains”-responsive gene set was significantly enriched in TF genes (*p*-value < 1.5 × 10^−12^) with the over-representation of the AP2/ERF-ERF, NAC, Tify, and WRKY TF families (corrected *p*-value < 0.05). Two expression groups were clearly identified including a set of 324 TF genes which were over-expressed in control samples in comparison with the infected ones and another set of 750 TF genes which were under-expressed in control samples in comparison with infected samples. TF genes belonging to the same family displayed similar expression patterns: the B3, B3-ARF, C2C2-CO-like, C2C2-YABBY, MADS_MIKC, mTERF, and Whirly families were mostly downregulated in response to FHB, while AP2/ERF-ERF, AP2/ERF-RAV, CAMTA, LOB, MYB, NAC, PLATZ, Tify, and WRKY families were mostly upregulated along with the infection progress (Fig. 4 and Additional file 1: Table S2).

### FHB induced the reprogramming of a wide array of biological processes

Downregulated genes in response to the three strains were enriched in 312 biological process (BP) subcategories (Additional file 1: Table S3A). Those categories were mainly associated with fundamental biological processes. Primary metabolism was widely impacted through several processes: photosynthesis (mainly through light harvesting), polysaccharide and monosaccharide synthesis, and starch metabolism, as well as amino acid and nucleotide synthesis. Cell and tissue structure was also altered at several scales including cytoskeleton and organelle structure as well as plasma membrane and cell wall. Several other cell basal functions were disturbed through the downregulation of genes involved in transduction, transport, protein sorting, transcription, and DNA repair and modification. Likewise, reproductive and cell cycle-related process were impacted and concerned 30% of the enriched BP terms including mitosis, meiosis, replication, chromosome separation, ovule, and embryo development and responses to auxin and cytokinin. Genes involved in terpenoid synthesis and in the negative regulation of the immune response were also downregulated along with the infection progress.

Upregulated genes in response to the three strains were enriched in 174 BP subcategories (Additional file 1: Table S3B). Genes involved in responses to several phytohormones, including gibberellin, salicylic acid, jasmonic acid, abscisic acid, cytokinin, and ethylene, were induced in response to the different *F. graminearum* strains. Genes involved in the synthesis of signaling specialized metabolites (chorismate, indolalkylamine, olefin, amine compounds, and oxylipin) were also over-represented. The biotic stress response associated categories (e.g., “systemic acquired resistance,” “receptor signaling,” “chitin catabolism”) as well as genes involved in the synthesis of several defense specialized metabolites (e.g., “sulfur containing compounds,” “phenylpropanoids,” “phenol compounds,” “trehalose,” and “prephenate”) were sharply activated along with the infection. The antioxidant machinery was also activated during the infection progress with the over-representation of genes involved in the reactive oxygen species (ROS) catabolism, in the detoxification processes, and in the synthesis of antioxidant specialized metabolites such as the glutathione and amine compounds. Genes associated with ion transport, calcium, ammonium, and metal ions as well as the trans-membrane transport of carbohydrates were upregulated. Cell integrity seems to be altered with an enrichment in the catabolism of several cell wall components: xylan, lignin, and amino-glycan. However, genes involved in the synthesis of cinnamic acid associated with the lignification process were also upregulated along with the infection progress as were the genes involved in pollen pistil interaction, protein ubiquitination and lipid catabolism.

### Establishing the transcription factor regulation network of FHB responses and searching for their master regulators

**Fig. 5:**
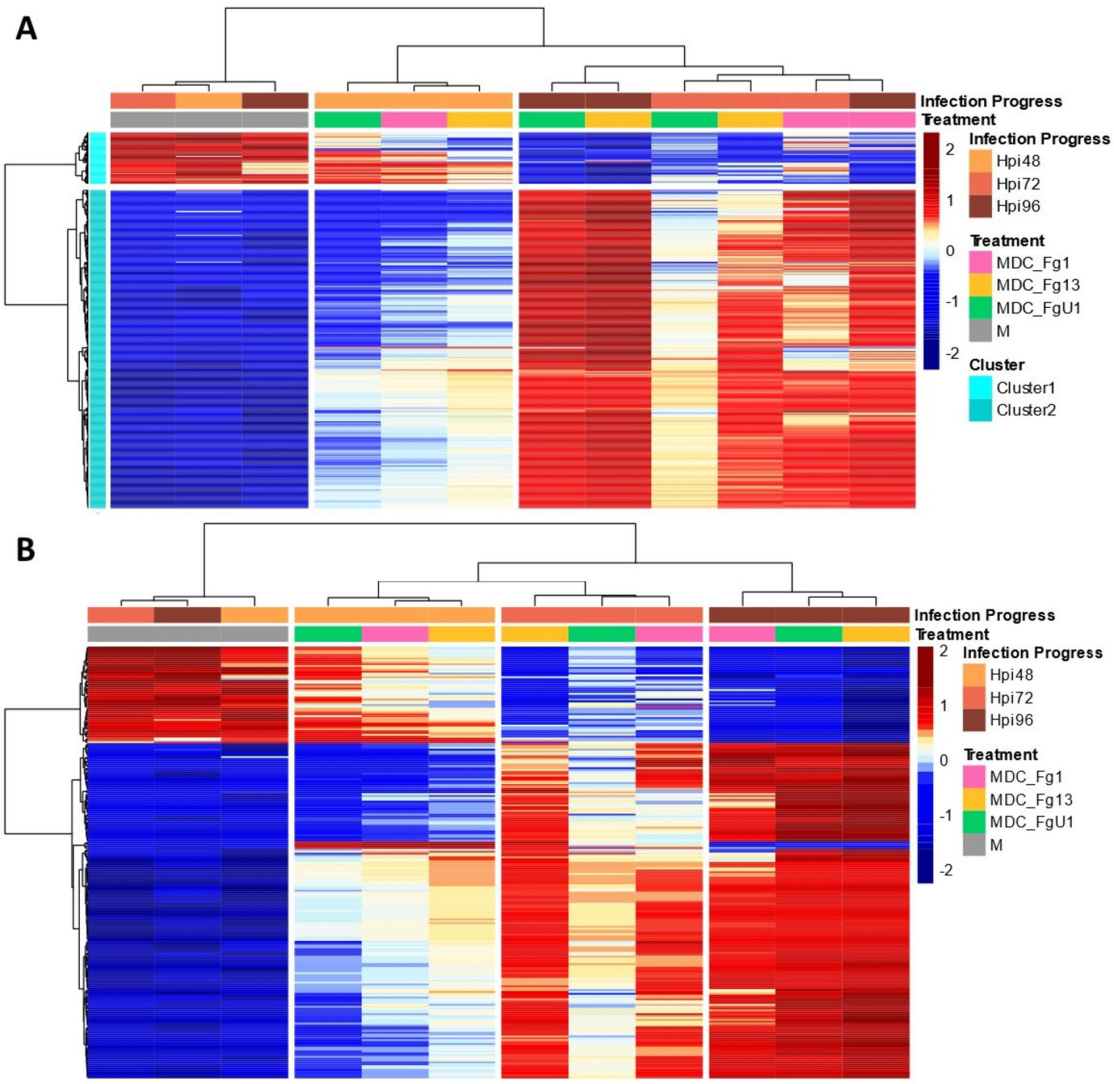
Expression regulation patterns of **A** the master TF regulating FHB response and **B** the 233 putative susceptibility genes targeted by *F. graminearum* nuclear effectors in “Recital” facing 3 strains of contrasting aggressiveness. The structure of gene and sample data sets were determined by HAC based on Ward’s minimum variance method using the *z*-score transformed gene expression values. Heatmap color scales represent the *z*-score transformed expression values of the genes for each condition. The clustering on top of the heatmap represents the experimental conditions which are labeled according to the factors infection progress and treatment. On the left side of the first heatmap (**A**), genes were colored according to their cluster membership

From the “All strains”-responsive gene set described in the single “Recital” susceptible cultivar facing the three different *F. graminearum* strains, we selected genes that were systematically expressed in the four other wheat cultivars (cv. “Arche,” “Courtot,” “Chinese Spring,” and “Renan”). As a whole, 20,629 genes (77% of the “All strains”-responsive gene set) were found in all cultivars regardless of their susceptibility level and were used to build the TF regulatory network of FHB responses. Among these genes, 772 were predicted to encode TFs. To establish the network, a set of 269 regulators were defined among which 16 corresponded to groups of highly correlated TFs (> 95% in absolute value). Statistical tests performed on the network edges led to a directional network composed of 9791 nodes and 28,581 edges (Additional file 3: R object S1). Within this TF regulatory network of FHB responses, we identified 91 master regulator nodes, i.e., a regulator node that regulated other regulator nodes without being regulated itself. Among these master regulator nodes, seven were groups of highly correlated TFs leading to a total of 427 master TFs (Additional file 1: Table S4). These master regulators belonged to 44 families, including four main families that gathered nearly half of the set, i.e., the WRKY (71 genes), the NAC (57 genes), the AP2/ERF-ERF (41 genes), and the MYB (40 genes) families. For example, TraesCS1B02G318800, encoding a MYB TF, had 34 targets including two TF genes, TraesCS6D02G362900 and TraesCS3D02G231200, which had 74 and 32 targets, respectively (Fig. 3D). Most of the master regulators were induced in response to the FHB infection (Fig. 5A, Additional file 1: Table S4); 368 master TF genes (cluster 2) were over-expressed in infected samples in comparison with controls, while only 59 (cluster 1) were downregulated during the infection process.

### Leveraging *F. graminearum* effector expression dynamics to identify susceptibility gene candidates

To identify putative susceptibility genes (S genes) targeted by *F. graminearum* effectors, we computed a second directional network using 46 nuclear core effectors of the three *F. graminearum* strains [49] as putative regulators of the 20,629 FHB responsive genes expressed in all wheat cultivars. Statistical tests performed on the edges led to a directional network composed of 17,393 edges connecting 41 nuclear effectors to 12,866 wheat targets (Additional file 3: R object S2), of which 6485 were also part of the TF regulatory network of FHB responses. FHB responsive genes which were not TFs represented 12,362 genes, of which 5981 were also found to be targeted by wheat TFs in the previous network. TF genes were significantly enriched (*p*-value < 0.05) within the effector regulation network, with a total of 504 genes targeted by *F. graminearum* nuclear effectors. Among them, 338 genes were identified as master regulators in the TF regulatory network of FHB responses.

As a whole, 233 wheat genes (Additional file 1: Table S5) targeted by *F. graminearum* effectors matched with known S genes [16, 31, 50], including 8 genes that were validated in the wheat—*F. graminearum* pathosystem [24, 26, 29, 51, 52]—and 183 genes that were associated with defense suppression. A total of 147 S genes were found to be part of the TF regulatory network of FHB responses, of which 41 were master TFs and 26 were TFs. Among the 41 master TF putative S genes, we found 33, 4, and 4 genes belonging to the WRKY, MYB, and NAC families, respectively. Among the 26 TFs that were not considered as master regulators, three families were found, including the MYB, WRKY, and EIL families that gathered 17, 7, and 2 genes, respectively.

### Expression signatures of the putative S genes in the susceptible wheat cultivar

As a whole, the 233 identified putative S genes were mostly induced in response to FHB, including 177 genes which were upregulated along with the infection progress and 4 genes over-expressed at 48 hpi only in infected samples (Fig. 5B and Additional file 1: Table S5) in comparison to control samples. Downregulated patterns in response to FHB were observed for 52 genes (Fig. 5B and Additional file 1: Table S5). Among the S genes involved in defense suppression, 149 genes were induced in response to FHB while 34 were downregulated. Regarding the master regulators, all the WRKYs and NACs as well as three of the four MYBs were induced in response to FHB while only one MYB was downregulated. Concerning the other TFs, all the WRKYs and EILs as well as 13 MYBs were also induced in response to FHB while only 4 MYBs were downregulated. For the non-TF S genes, 115 were induced along with the FHB progress with the maximum of expression reached at 96 hpi, four were over-expressed in infected samples at 48 hpi, and 47 were over-expressed in controls.

### Did putative FHB susceptibility genes discriminate different wheat genetic background?

**Fig. 6:**
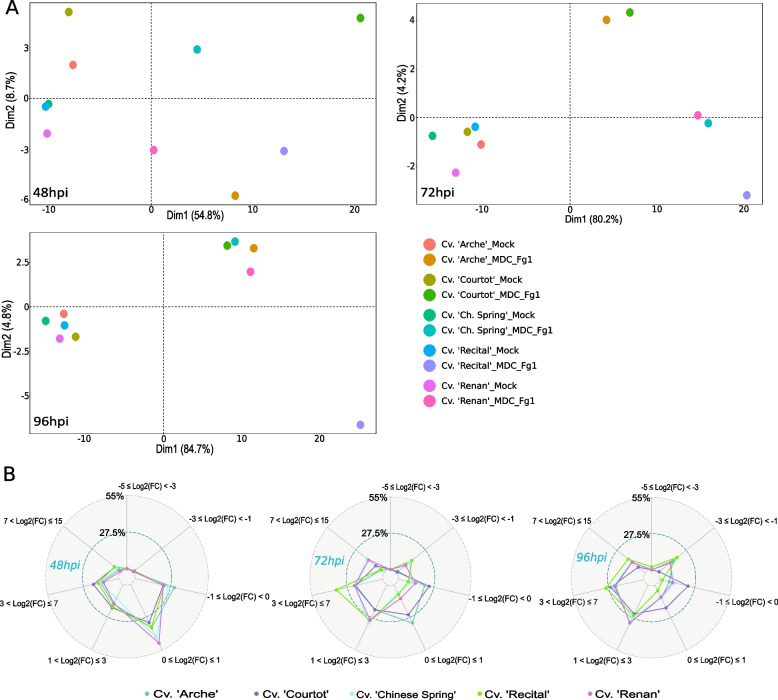
FHB responses of the five cultivars on the 233 putative S genes at 48 hpi, 72 hpi, and 96 hpi. **A** PCAs were computed on the *z*-score values of the S gene set at each time point. The plots of the individuals extracted from the PCAs are represented on the two first components, on which statistical tests were performed. **B** For each time point, a spider chart describes the log2(FC) of the S genes between infected and control conditions in “Arche,” “Courtot,” “Chinese Spring,” “Recital,” and “Renan.” Colored points indicate the proportion of genes belonging to each of the seven log2(FC) ranges in each cultivar. Extreme values were the maximum and minimum log2(FC) of all comparisons. A negative log2(FC) indicates a lower expression level in the infected condition than in the control one and a positive log2(FC) indicates a higher expression level in infected condition than in the control one. Spider charts are composed of two grid circles, the middle grid circle indicating 27.5% and the maximum grid circle indicating 55%

To identify specific cultivar’s FHB responses, we performed a principal component analysis (PCA) on the S gene set for each time point (Fig. 6A) as well as statistical tests on their components. For all the 3 time points, the first component, which explained 54.8%, 80.2%, and 84.7% of the variance at 48 hpi, 72 hpi, and 96 hpi respectively, significantly discriminated the infected samples from the control ones. At 48 hpi, the position of the infected “Courtot” responses on the component 1 was significantly different from infected “Arche,” “Chinese Spring,” and “Renan” responses, and the position of infected “Recital” was significantly different from infected “Renan” responses. At 48 hpi, the second component (8.7%) was significantly driven by the infected “Arche” responses. At 72 hpi, the position of infected “Recital” responses on the component 1 was significantly different from infected “Arche” and “Courtot” responses, and the position of infected “Chinese Spring” was significantly different from infected “Arche” responses. At 72 hpi, the second component (4.2%) significantly distinguished the infected samples of “Arche” and “Courtot” from the infected samples of “Recital.” At 96 hpi, the position of infected “Recital” on component 1 was significantly different from all other infected cultivars, while the component 2 (4.8%) significantly discriminated infected condition from the control one for all cultivars as well as the infected “Recital” from all other infected cultivars.

The magnitudes of FHB responses were different between the 5 cultivars (Fig. 6B). This can be notably observed at the low FC ranges. At 48 hpi, “Renan,” “Chinese Spring,” and “Arche” displayed a relatively high proportion of low FC responses (− 1 ≤ *Log2*(FC) ≤ 1), including 71.24%, 69.96%, and 66.95% of the S genes respectively. Such low FC responses only represented 58.8% and 54.08% of the S genes in “Recital” and “Courtot” respectively. At 72 hpi, “Arche” and “Courtot” displayed 52.8% and 49.36% of the S genes with a *Log2*(FC) between − 1 and 1, while it accounted for only 24.9%, 17.17%, and 15.88% in “Renan,” “Chinese Spring,” and “Recital.” At 96 hpi, we distinguished two opposite patterns of responses with “Courtot” displaying 41.63% of the S genes with a *Log2*(FC) between − 1 and 1 while “Recital” displayed 93.13% of the S genes with a *Log2*(FC) above 1 or below − 1. “Chinese Spring,” “Renan,” and “Arche” displayed 23.18%, 20.6%, and 18.45% of the S genes with a *Log2*(FC) between − 1 and 1. In the HostV experiment, 142 out of the 233 putative S genes displayed significant differences between control and infected samples for all 5 cultivars, of which 119 were involved in defense suppression and 53 encoded TFs. Almost all those 142 conserved S genes, except 5 genes, were over-expressed along infection progress in all 5 cultivars (Additional file 2: Fig. S2). With a total 224 DEGs in the HostV experiment, “Recital” displayed the highest number of FHB responsive DEGs. “Chinese Spring,” “Arche,” “Renan,” and “Courtot” respectively displayed 215, 193, 171, and 159 FHB responsive DEGs.

### Genomic localization of the putative FHB susceptibility genes

The distribution of the S genes on the wheat genome was balanced at the sub-genome level with 77, 76, and 77 genes localized on the sub-genomes A, B, and D, respectively (Additional file 2: Fig. S3). S genes were also localized over all the 21 wheat chromosomes (Table 1). Localization on the unknown chromosome accounted for three genes. A total of 2 and 1 genes were found within the susceptibility intervals validated by Garvin et al. [21] and by Hales et al. [22], respectively. A total of 27 S genes, including two genes validated in the wheat–*F. graminearum* pathosystem, TaHRC [51] and TaEIN2 [52], were also found within the 118 known FHB meta-QTLs (13; Additional file 2: Fig. S3) on 16 different chromosomes. Among the 27 S genes, six genes including three WRKYs, two NACs, and a single MYB were identified as master regulators in the TF regulatory network of FHB responses. The 21 other genes were not TF genes. All master TF genes, except the MYB TF gene, were over-expressed in infected samples in comparison to the control samples. Regarding the non-TF genes, 13 genes were over-expressed in infected samples in comparison to the controls while 8 genes were under-expressed.

**Table 1:** S gene distribution on the wheat genome

**Chromosome**	**S gene number**
chr1A	11
chr1B	10
chr1D	15
chr2A	13
chr2B	13
chr2D	13
chr3A	11
chr3B	9
chr3D	9
chr4A	15
chr4B	15
chr4D	12
chr5A	11
chr5B	10
chr5D	9
chr6A	8
chr6B	10
chr6D	8
chr7A	8
chr7B	9
chr7D	11
chrUn	3

## Discussion

Using *F. graminearum* strains of contrasting aggressiveness, we searched for consistent FHB induced transcriptional responses that are supposed to drive the infection process in ‘Recital,” a susceptible wheat cultivar. Gene regulatory network modeling as well as the responses from cultivars of different susceptibility to FHB were then leveraged to thoroughly characterize the regulation processes as well as to identify robust susceptibility genes that might be targeted by *F. graminearum*.

### Wheat response to FHB is driven by specific master regulators that control a complex gene regulation network

Transcriptome profiling of “Recital” responses to the three *F. graminearum* strains of contrasting aggressiveness revealed an early and sharp alteration of wheat spike basal functioning through the downregulation of genes involved in primary metabolism, in spike development and in cell structure, while genes involved in signaling pathways, biotic stress responses, and detoxification processes were steeply induced from the infection outset. The control of “Recital” transcriptome reprogramming in response to FHB involves a large number of TFs and TF families. Examining the relationship between these regulators showed that FHB-responsive TFs displayed highly correlated expression patterns and evidenced major regulation hubs of the FHB responses. Four TF families, the WRKY, NAC, AP2/ERF, and MYB families, that represented a total of 209 master regulator gene candidates induced along infection progress, were distinguished for their central role in FHB responses. Those four families are well-known key players of biotic stress response that can positively or negatively affect the setup of defense mechanisms contributing to either resistance or susceptibility [33, 53–55]. In wheat resistant lines facing *F. graminearum*, two NAC TFs, the TaNAC032 [56], and the TaNACL-D1 [57], as well as one WRKY TF, the TaWRKY70 [58], were already been associated with the setup of defense mechanisms, such as in lignin synthesis and in resistance-related induced (RRI) metabolite production. On the opposite, the two NAC TFs, TaNAC2 [59] and TaNAC30 [60], were shown to negatively regulate ROS accumulation and to increase wheat susceptibility to *Puccinia striiformis*. In line with our results, those TF families were proved to be organized in complex regulation networks involving autoregulatory processes, including intra-family cross-regulatory processes [54, 61–64] and inter-family cross-regulatory processes [65, 66]. This concerted transcriptional modulation could represent an efficient way to target multiple biological processes and to set up fine-tuned responses to the fungal infection.

### Master regulators of FHB responses: targets of fungal core effectors and key players of FHB susceptibility

Integrating expression data from both protagonists of the interaction highlighted that transcription factors and especially master regulators could represent main direct or indirect targets of *F. graminearum* core nuclear effectors. As in the transcription network of FHB responses, we found that the four most represented families among the expected targeted TFs were the WRKY, NAC, MYB, and AP2/ERF families, strengthening their prime role in regulating FHB responses. Those families were previously shown to be upregulated in the “Shaw” susceptible wheat cultivar in response to *F. graminearum* [42]. TF manipulation by direct or indirect action of effectors were described in many plant-fungus interactions. Direct interactions between effectors and plant TFs were described in the symbiotic fungi *Glomus intraradices* that secretes the SP7 effector which interact with the AP2/ERF19 TF of *Medicago truncatula* in order to deactivate defense responses [67] but also in the hemibiotrophic fungi *Verticillium dahliae* with the VdSCP41 effector that targets the two transcription factors CBP60g and SARD1 in *A. thaliana* to alter their immunity functions [68]. An example of indirect effect on a plant TF was described in the biotrophic pathogen *Ustilago maydis* that secretes the Tin2 effector which prevents the degradation of the ZmTTK1 protein kinase which in turn triggers the nuclear addressing of the ZmR1 maize TF [69].

### Hijacking of plant defense and immune responses, a conserved strategy determining FHB susceptibility in wheat

**Fig. 7:**
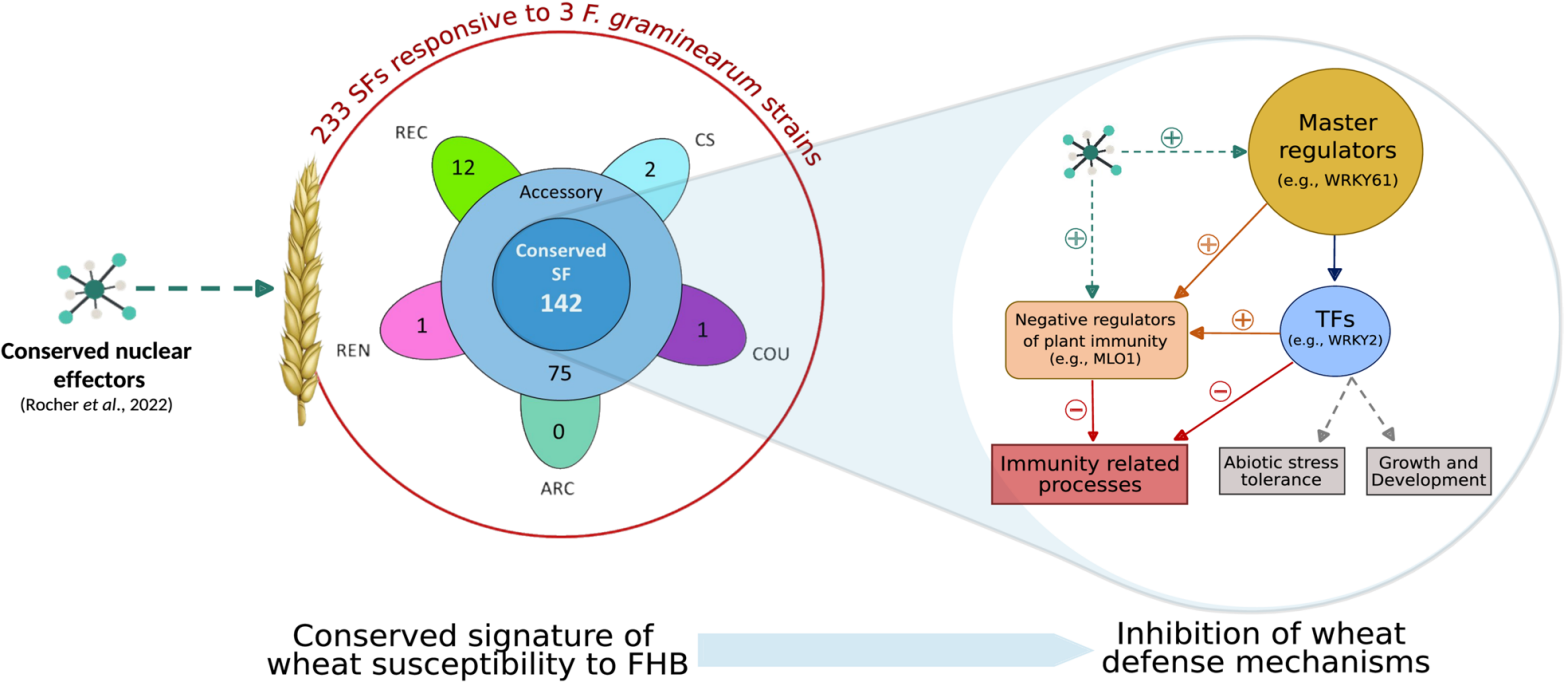
Representation of the susceptibility mechanism underlying the wheat–*F. graminearum* interaction. Integrating the expression data from both wheat and *F. graminearum* as well as using the genetic variability from both species allowed to identify 142 conserved susceptibility genes. The manipulation of the regulation hubs as well as negative regulators of plant immunity in order to suppress the defense response appeared to be a key step in the infectious process of* F. graminearum*

Merging the S genes database and the *F. graminearum in planta* targets, we identified 233 S genes putatively targeted by *F. graminearum* core nuclear effectors (Fig. 7), of which 64% were induced along with the infection progress and were proved to have a role in defense suppression with a wide range of mechanisms [16, 31, 50]. Interestingly, our integration approach shed light on 8 genes already validated as S genes in the wheat–*F. graminearum* pathosystem including the TaHRC gene that underlies the Fhb1 QTL [27, 28] and that was induced in response to *F. graminearum* infection. The multi-gene model underlying the Fhb1 QTL points out that TaHRC [27, 28] operates as a susceptibility factor through the inhibition of the calcium-mediated defense response [51] and that its activity leads to the repression of the WFhb-1 gene involved in antifungal activity [70]. Further evidences in the wheat–*F. graminearum* strategy also support the key role of defense suppressor genes in the compatibility of the interaction, such as the induction of several genes found to be nuclear effector targets, TaSSI2 [26], TaLpx-1 [24], and TaNFXL1 [29], which are involved in the suppression of salicylic acid (SA)-mediated defenses. In this study, we also identified nine Mildew resistance locus O (Mlo) gene homologues that were upregulated upon FHB infection. Encoding a membrane protein involved in the negative regulation of immunity, Mlo is a well-characterized susceptibility gene in barley and wheat to the biotroph pathogen *Blumeria graminis* and its KO mutation confers a broad-spectrum resistance [71–73]. Resistance through the silencing of Mlo genes was also reported in pathosystems involving necrotrophs such as in the Cucumis sativus–*Corynespora cassiicola* interaction [74]. Six more upregulated genes matching the DMR6 gene of *A. thaliana* suggest another mechanism of defense suppression through the degradation of salicylic acid (SA), known to trigger the defense responses against biotrophic and hemibiotrophic pathogens [75, 76]. Because the *A. thaliana* DMR6 gene and its barley orthologue were proved to promote FHB susceptibility [77], those six wheat genes represent reliable candidates S genes for further functional validation. Furthermore, TFs were also largely represented among the putative S genes involved in defense suppression with 64 TFs genes (96% of the effector targeted TF genes with a match in the S genes database) induced along infection progress, belonging to the WRKY (38 genes), NAC (4 genes), MYB (20 genes), and EIL (2 genes) families. For instance, they included the three TaWRKY61 homoeologues validated in the wheat–*Puccinia striiformis* interaction that are positive regulators of the TaSTP3 sugar transporter, found among our targets, and whose induction in response to infection results in cytoplasmic sugar accumulation and suppression of defense-related genes [78]. By interacting with TF genes, *F. graminearum* appears to repress pivotal components of cell immunity, and in this respect, it could be able to manipulate at a systemic scale a wide array of defense-related processes (Fig. 7). This large number of putative targeted wheat genes appears consistent with the highly complex and fragmented nature of the FHB resistance controlled by 625 QTLs distributed throughout the genome [12, 13]. All the meta-QTLs, within which were localized our putative S genes, were associated with spreading within the spike, and 16 were also associated with mycotoxin accumulation [13]. Taken together, these evidences indicate that *F. graminearum* targets several defense pathways and partly suppresses plant defense mechanisms to promote its successful establishment and development in the spike. This infection strategy deduced from the plant responses was further demonstrated to be highly conserved in response to the three *F. graminearum* strains. This finding fully corroborates previous results reporting that the same three strains shared 90% of their *in planta*-established effectome [49].

While *F. graminearum* was able to infect the 5 wheat cultivars, only 61% of the S genes were significantly regulated in all the cultivars. Thus, pathogen’s establishment in cultivars of contrasting susceptibility appeared to rely on a conserved corpus of S genes successfully manipulated in all wheat genetic backgrounds. The most susceptible wheat cultivar “Recital” was clearly discriminated from the 4 others in terms of DEG number and response amplitude. However, there were no clear link between the susceptibility level of the 4 other cultivars and their differences observed at the transcriptomic level. This exemplifies the complex nature of wheat–*F. graminearum* interaction and might highlight some redundancy in the processes targeted by *F. graminearum* effectors. Taking advantage of different wheat cultivars with contrasting susceptibility level, this provides a robust list of 142 susceptibility gene candidates, including 119 defense suppressors and 53 TF genes, that responded to several *F. graminearum* strains and systematically regulated in different wheat genetic backgrounds. Since almost all these conserved S genes were induced in response to infection, the suppression of plant immunity appears to be a pivotal step in *F. graminearum* establishment success regardless of the fungal strain and the wheat cultivar (Fig. 7).

## Conclusions

To conclude, using a system-level transcriptomics data integration and mining genetic variability on both host and pathogen sides, we discovered unexpected conserved responses of wheat facing FHB. This portrays a distinctive signature of the intricate mechanisms that drive the outcome of the wheat–*F. graminearum* interaction and further enabled to delineate a set of core-transcription factors putatively involved in the orchestration of susceptibility factors. Such robust conservation emphasizes that wheat susceptibility to FHB could be one of the main drivers of disease establishment and, as such, reshapes the way we consider plant responses to pathogens beyond the role and expression of the defense genes alone. In addition to their involvement in FHB, the identified candidate S genes could conceivably be of genetic value in controlling a broader spectrum of pathogens [17, 79, 80], providing original clues for further researches.

## Methods

### Experimental designs and plant sample production for transcriptomic analyses

To perform a comprehensive characterization of wheat responses to FHB, a first experiment, referred to as PathoV, was conducted on the susceptible wheat cultivar “Recital” which was inoculated with three *F. graminearum* strains of contrasting aggressiveness (MDC_Fg1, MDC_Fg13 and MDC_FgU1; in decreasing order of aggressiveness [81]) or with water as control. To study the FHB responses of different wheat genetic backgrounds, a second experiment referred to as HostV was conducted on five cultivars of contrasting susceptibility, “Arche” (ARC), “Courtot” (COU), “Chinese Spring” (CS), “Recital” (REC), and “Renan” (REN); all were inoculated with the aggressive strain MDC_Fg1 or with water as control. The two experiments were conducted to collect samples at 48, 72, and 96 h post inoculation (hpi), which cover a pre-symptomatic period and the symptom onset stage [45]. Spore production as well as detailed plant growth conditions are described in Rocher et al. [49]. Complete factorial experiments were designed for both experiments. For the first experiment, the combinations of the four treatment conditions (three strains + one control) and the three time points were measured on four biological replicates (except for MDC_Fg13 × 72 hpi condition with three replicates) that represented a total of 47 samples. For the second experiment, the combinations of the five cultivars, the two treatment conditions, and the three time points were measured on three biological replicates that represented a total of 90 samples. Each experiment was surrounded by additional plants to limit any edge effect. *F. graminearum* strains were inoculated at mid-anthesis with 10 μL of a solution at a concentration of 10^5^ spores/mL in the floral cavity of the six central spikelets of three synchronous flowering spikes (totaling 18 spikelets per biological replicate). For the mock samples, water was inoculated according to the same procedure. For each individual plant, only the inoculated spikelets were collected and immediately placed in liquid nitrogen. Samples were ground in fine powder and stored at − 80 °C before RNA extractions.

### RNA extraction and sequencing

The detailed procedures have already been described in Rocher et al. [49]. From 100 mg of frozen powder, total RNA extraction was performed using a TRIzol protocol (TRI reagent®, Sigma-Aldrich, St. Louis, MO, USA), followed by a FastDNase treatment (TURBO DNA-freeTM Kit, Thermo Fisher Scientific, Waltham, MA, USA). Sample quality was controlled by electrophoresis with 1% agarose gel buffered in Tris–Acetate-EDTA. A total of 10 μg per sample was used for sequencing. The cDNA libraries were produced using the TruSeq Stranded preparation kit reverse oriented (Illumina, San Diego, CA, USA). For both experiments, 2 × 150 base paired-end sequences were generated. Samples of the first experiment dealing with the single cultivar “Recital” were sequenced using Illumina NovaSeq6000 at GeT platform of GenoTOUL [82], and the samples of the second experiment dealing with the five wheat cultivars were sequenced using Illumina HiSeq4000 and NovaSeq6000 at the Genoscope, the French National center of sequencing [83].

### RNA-Seq bioinformatic analysis

Sequencing data obtained from both PathoV and HostV experiments were analyzed separately, using a host–pathogen genome mapping approach. Calculations were performed on the supercomputer facilities of the Mésocentre Clermont Auvergne University [84] and on the TGCC infrastructure of the CEA [85]. The construction of *F. graminearum* pangenome used in the mapping step is fully described in Rocher et al. [49].

From raw reads, TrimGalore v0.6.5 [86] was used to remove the adapters and low-quality bases (phred score < 20), while homemade Perl scripts were developed to trim the non-called bases and the polyA tails. Reads exhibiting a low complexity level (compression size < 65%) and a short size (size < 60 nucleotides) were discarded using homemade Perl scripts. A decontamination step was performed by mapping with STAR v2.7.1a [87] against a homemade database of potential contaminants composed of 1649 viral genomes, 9267 bacterial genomes, and the human genome, downloaded from the NCBI Reference Sequence Database [88]. The trimming of ribosomal RNA reads was performed with SortMeRNA v4.2.0 [89] against a database of wheat and *F. graminearum* rRNAs built from noncoding RNA genes fasta files of the *Triticum aestivum* v1.0 (GCA_900519105.1) assembly [90] and PH-1 (GCA_900044135.1) genome assembly [91]. Genome and annotation files of *Triticum aestivum* [90, 92] and *F. graminearum* pangenome were merged into a host–pathogen genome containing 269,428 (high-confidence and low-confidence genes) wheat genes and 17,647 *F. graminearum* genes. The mapping against this combined genome was performed using STAR v2.7.1a. Considering only the uniquely mapped read pairs, gene-level counts were generated for each species using the featureCounts software of the subread v2.0.1 package [93].

### Statistical analysis of wheat expression data

#### Differential expression analyses

Statistical analysis was conducted on R v4.1.1 [94]. For the PathoV experiment, genes were filtered per treatment modality using a 4 counts per million (CPM) threshold in at least 3 samples independently of the time point. This resulted in an expression matrix of 49,505 genes. For each cultivar of the HostV experiment, genes were filtered per treatment modality with a 4 CPM threshold in at least 3 samples independently of the time point. Expression matrices of “Arche,” “Courtot,” “Chinese Spring,” “Recital,” and “Renan” were composed of 45,181; 44,416; 46,306; 46,120; and 45,252 genes respectively. For both experiments, expression matrices were normalized according to library size with the trimmed mean of *M* values (TMM) method implemented in edgeR package [95, 96]. DE analyses were performed using a negative binomial generalized linear model. DE analyses were conducted using the DiffAnalysis_EdgeR function of the DicoExpress script-based tool [97], based on EdgeR package and generating all the contrasts automatically for complex experimental designs. For the PathoV experiment, the log2 of the mean normalized gene expression is an additive function of a treatment effect (4 modalities), a time effect (3 modalities), and an interaction between the treatment and the time (12 modalities). A total of 48 contrasts were considered, which were defined for 12 of them as the difference between two time points given a treatment (kinetic effect), for 18 of them as the difference between two treatments given a time point (treatment effect) and for the last 18 as the interaction term, i.e., the difference between two time points given a treatment minus the difference between the same two time points given another treatment (interaction effect). For the HostV experiment, a model was designed for each cultivar where the log2 of the mean normalized gene expression is an additive function of a treatment effect (2 modalities), a time effect (3 modalities), and an intersection between the treatment and the time (6 modalities). A total of 12 contrasts were considered for each cultivar. They were defined for six of them as the difference between two time points given a treatment (kinetic effect), for three of them as the difference between infected and mock samples given a time point (treatment effect), and for the last three as the difference between two time points in infected samples minus the difference between the same two time points in mock samples (interaction effect). A likelihood ratio test was performed on each contrast and the false discovery rate was controlled with the Benjamini–Hochberg adjustment procedure of the raw *p*-values [98]. An adjusted *p*-value threshold of 0.001 was set to declare a gene differentially expressed. The base 2 logarithm of fold changes (*Log2*FC) were visualized with spiderplots generated using the ggradar R package [99].

#### Expression pattern characterization

For the HostV experiment, filtered expression matrices from each cultivar have been merged into a single expression matrix. Prior to the analyses and for both experiments, filtered raw count values were normalized using the regularized logarithm transformation (rlog) implemented in the DESeq2 package [100]. For all the following analyses, a *z*-score transformation was applied to the normalized count values. Gene expression patterns were described with heatmaps using the Pheatmap package [101]. The genes and samples were clustered using the ward.D2 agglomeration method applied on the Euclidean distance matrices [102]. To assess the capacity of the selected genes to classify the infected samples from the PathoV experiment according to the strain × time point combinations, a PLS-DA was performed using the R package mixOmics [103] with 10 components and default parameters for the other options. To characterize the cultivars responses to FHB, PCA analyses at each time point were computed using the FactoMineR R package [104]. The coordinates of the individuals on the two first PCA’s components were retrieved and were used to compute a complete linear model for each component, where the position on the component is an additive function of a cultivar effect (five modalities), a treatment effect (two modalities), and an interaction between the two factors (ten modalities). A *p*-value threshold of 0.05 was applied.

#### Regulation networks computation

The TF regulation network of FHB responses and the effector regulation network were computed using the network_inference function of the DIANE R package [105] based on the GENIE3 R package [106]. For both networks, the rlog normalized counts of the selected genes were used as input expression values. For the TF regulation network of FHB responses, since regression methods are very sensitive to correlations between regulator genes, the regulator genes displaying a spearman correlation above 95% in absolute value were grouped using the group_regressors function of DIANE package. For each group of highly correlated regulators, a new expression value is computed using only the average expression value of the positively correlated genes. For the effector regulation network, no grouping based on correlations was applied. For both networks, in order to infer connections between regulator and target genes, random forests machine learning method was applied on the gene expression profiles to determine the influence of each regulator gene on each target gene. In the TF regulation network of FHB responses, the regulator genes were also part of the target gene set. For both networks, we applied the following procedure to select the most robust gene pairs without using a hard-threshold on edge weights. The edge’s importance matrix was computed using the “MSEincrease_oob” option as importance metric in the network_inference function. Then, to statistically test the significance of each edge, the test_edges function was applied on the inferred networks with the following settings: density = 0.02, nTrees = 1000, nShuffle = 1000. Edge importance *p*-values were adjusted according to the Benjamin-Hochberg procedure. The final networks were built from edges with an adjusted *p*-value below 0.02 using the network_from_tests function. Network resume tables were created using the network_data function, which also performed the Louvain algorithm [107] that defines communities of nodes within the networks. Network visualization was performed using the visNetwork R package [108].

#### Wheat gene ontology and annotations

Gene Ontology (GO) terms of *Triticum aestivum* v1.1 genome annotation [92] were retrieved from EnsemblPlants using the BiomaRt R interface [109]. GO terms were then used to build a wheat annotation orgdb R package using the makeOrgPackage function of the AnnotationForge R package [110]. To predict transcription factors, the iTAK software [111] was applied on the high (HC)- and low-confidence (LC) wheat proteomes v1.1. To use only the most robust TF genes in the subsequent analysis, we kept the intersection between the TF gene set predicted by iTAK and the high-confidence TF gene set identified in Ramirez et al. [112].

A database of susceptibility factors was built using known susceptibility genes of wheat listed in [31, 50] leading to a first database of 132 wheat susceptibility factors. To complete this database, the protein sequences of the susceptibility genes validated in other plant species, listed in [16], were retrieved from the UniProt Knowledgebase [113], leading to a second database of 185 susceptibility factors. Their homologues in wheat HC and LC proteomes were searched using Blastp [114] with an identity threshold of 60%.

All enrichment analysis were performed using a hypergeometric test. Enrichment in TF genes and TF families in the “All strains”-responsive gene set were computed against the PathoV expressed TF gene set using the phyper function of the R stats package [115] with a *p*-value threshold of 0.05. Enrichment in TF genes within effector’s targets was performed against the TFs genes used in the input of the effector regulation network using the same method as for the TFs of the “All strains”-responsive gene set. Functional enrichment analysis of the “All strains”-responsive gene set was conducted against the PathoV expressed genes, using the enrichGO function of the clusterProfiler v4.0 R package [116] on wheat’s Biological Process GO terms. Benjamin-Hochberg procedure was used to adjust the *p*-values. An adjusted *p*-value threshold of 0.05 was set to declare over-represented a BP category.

#### Genomic localization of the putative susceptibility genes

In order to identify S genes that colocalize within loci associated with FHB resistance, genomic localizations of the identified S genes retrieved from the Gene Transfer Format (GTF) annotation file of wheat and the positions of the FHB resistance meta-QTLs in wheat, downloaded from the supplementary materials published in Zheng et al. [13], were plotted on wheat genome using the karyoplotR R package [117].

#### Score symptom monitoring experiment and analysis

To evaluate the susceptibility level of the wheat cultivars used in HostV, a complete factorial experiment was repeated in the same growth chamber than HostV and PathoV, from three additional biological replicates per cultivar. MDC_Fg1 spores were inoculated as for HostV and PathoV, at mid-anthesis with 10 μL of a solution at a concentration of 10^5^ spores/mL in the floral cavity of the six central spikelets of three synchronous flowering spikes of each biological replicate. The symptoms were scored for each inoculated spikelet using a non-destructive 5-level rating scale, as described in Fabre et al. [45, 81]. The symptoms produced by MDC_Fg1 on the five wheat cultivars were evaluated every 24 h from 48 to 168 hpi. The statistical analysis was conducted on the score symptoms averaged per biological replicate. As described in Gilligan [118], the progress of symptom development was modelized using logistic regression curves using the following equation:

$$f\left(x\right)=\frac{Asym}{1+e\left(\frac{-x-xmid}{scal}\right)},$$

where *x* is the duration in hpi nested in the biological replicates, *Asym* is the asymptote that modelized the maximum score symptom reached at the end of the experiment, *xmid* is the time required to reach 50% of the *Asym* score, and *scal* is the time required to switch from 50 to 75% of the *Asym* score. Modelization was performed using the nlsList function with the SSlogis setting from the nlme R package [119]. *Asym*, *xmid*, and *scal* parameters were retrieved for each plant and were analyzed with a one-way ANOVA to test the cultivar effect. To refine the susceptibility ranking of the 5 cultivars, a hierarchical ascendant clustering (HAC) was performed with the complete linkage method applied on the Euclidean matrix computed from the scaled regression’s parameters.

### Supplementary Information

**Additional file 1: Table S1.** Resume table of the statistical analysis performed on 'Recital' genes when facing one of the three different *F. graminearum* strains. **Table S2.** Expression patterns for each identified TF family that were FHB-responsive in 'Recital' facing one of the three *F. graminearum *strains. **Table S3.** A GO BP term enrichment results on the gene set under-expressed in FHB samples compared with controls. B GO BP term enrichment results on the gene set over-expressed in FHB samples compared with controls. **Table S4.** Resume Table of the master regulators identified in the TF regulatory network of the FHB responses. **Table S5.** Resume table of the identified known S genes.

**Additional file 2: Fig. S1.** Expression regulation patterns of the ‘All strain’ -responsive gene set in ‘Recital’ along the time course for control and infected samples. **Fig. S2.** Expression regulation patterns of the 142 susceptibility genes targeted by *F. graminearum* nuclear effectors that are differentially expressed in response to FHB in the 5 wheat cultivars (HostV). **Fig. S3.** Genomic distribution of the 233 putative S genes on wheat subgenomes A (S3A), B (S3B) and D (S3C).

**Additional file 3.** R objects S1-S2. R object S1—TF regulation network FHB responses in recital. R object S2—Nuclear effector regulation network

## Data Availability

All data generated and analyzed for this study are included in this published article, supplementary files, and publicly available platforms. PathoV RNA-seq raw reads (NewMyco project) that support these findings were deposited as fastq.gz files in the European Nucleotide Archive (ENA) database, project reference number PRJEB59062 [120]. HostV RNA-seq raw reads (WheatOMICS project) that support these findings were deposited in the ENA database, project reference number PRJEB59238 [121].

## Abbreviations

FHB: Fusarium head blight
DON: Deoxynivalenol
QTLs: Quantitative trait loci
S genes: Susceptibility genes
TF: Transcription factor
RNAseq: mRNA sequencing
hpi: Hours post inoculation
Asym: Maximum score symptom reached at 168 hpi
xmid: Time required to reach 50% of this maximum score
scal: Time required to switch from 50 to 75% of the maximum score symptom
HAC: Hierarchical ascending clustering
DE: Differential expression
DEG: Differentially expressed gene
M: Mock
FC: Fold change
PLS-DA: Partial least square discriminant analysis
BP: Biological process
ROS: Reactive oxygen species
PCA: Principal component analysis
SA: Salicylic acid
PathoV: Pathogen strain variability experiment
HostV: Wheat host variability experiment
ARC: ‘Arche’ wheat cultivar
COU: ‘Courtot’ wheat cultivar
CS: ‘Chinese Spring’ wheat cultivar
REC: ‘Recital’ wheat cultivar
REN: ‘Renan’ wheat cultivar
rRNA: Ribosomal RNA
CPM: Counts per million
TMM: Trimmed mean of *M* values
Log2FC: Base 2 logarithm of fold change
rlog: Regularized logarithm transformation
GO: Gene Ontology
HC: High confidence
LC: Low confidence
GTF: Gene Transfer Format
ENA: European Nucleotide Archive

## References

[1] Parry DW, Jenkinson P, McLeod L (1995). Fusarium ear blight (scab) in small grain cereals—a review. Plant Pathol.

[2] Goswami RS, Kistler HC (2004). Heading for disaster: Fusarium graminearum on cereal crops. Mol Plant Pathol.

[3] Boyacioǧlu D, Hettiarachchy NS (1995). Changes in some biochemical components of wheat grain that was infected with Fusarium graminearum. J Cereal Sci.

[4] Argyris J, Sanford D, TeKrony D (2003). Fusarium graminearum infection during wheat seed development and its effect on seed quality. Crop Sci.

[5] Chen Y, Kistler HC, Ma Z (2019). Fusarium graminearum trichothecene mycotoxins: biosynthesis, regulation, and management. Annu Rev Phytopathol.

[6] McMullen M, Bergstrom G, De Wolf E, Dill-Macky R, Hershman D, Shaner G (2012). A unified effort to fight an enemy of wheat and barley: Fusarium head blight. Plant Dis.

[7] Dahl B, Wilson WW (2018). Risk premiums due to Fusarium head blight (FHB) in wheat and barley. Agric Syst.

[8] Wilson W, Dahl B, Nganje W (2018). Economic costs of *Fusarium* head blight, scab and deoxynivalenol. World Mycotoxin J.

[9] Vaughan M, Backhouse D, Ponte EMD (2016). Climate change impacts on the ecology of Fusarium graminearum species complex and susceptibility of wheat to Fusarium head blight: a review. World Mycotoxin J.

[10] Mylonas I, Stavrakoudis D, Katsantonis D, Korpetis E, Ozturk M, Gul A (2020). Chapter 1 - better farming practices to combat climate change. Climate change and food security with emphasis on wheat.

[11] Xia R, Schaafsma AW, Wu F, Hooker DC (2020). Impact of the improvements in Fusarium head blight and agronomic management on economics of winter wheat. World Mycotoxin J.

[12] Venske E, dos Santos RS, da Farias DR, Rother V, da Maia LC, Pegoraro C (2019). Meta-analysis of the QTLome of Fusarium head blight resistance in bread wheat: refining the current puzzle. Front Plant Sci.

[13] Zheng T, Hua C, Li L, Sun Z, Yuan M, Bai G (2021). Integration of meta-QTL discovery with omics: towards a molecular breeding platform for improving wheat resistance to Fusarium head blight. Crop J.

[14] Vogel JP, Raab TK, Schiff C, Somerville SC (2002). PMR6, a pectate lyase–like gene required for powdery mildew susceptibility in Arabidopsis. Plant Cell.

[15] O’Connell RJ, Panstruga R (2006). Tête à tête inside a plant cell: establishing compatibility between plants and biotrophic fungi and oomycetes. New Phytol.

[16] van Schie CCN, Takken FLW (2014). Susceptibility genes 101: how to be a good host. Annu Rev Phytopathol.

[17] He Q, McLellan H, Boevink PC, Birch PRJ (2020). All roads lead to susceptibility: the many modes of action of fungal and oomycete intracellular effectors. Plant Commun.

[18] Jaswal R, Kiran K, Rajarammohan S, Dubey H, Singh PK, Sharma Y (2020). Effector biology of biotrophic plant fungal pathogens: current advances and future prospects. Microbiol Res.

[19] Jørgensen IH (1992). Discovery, characterization and exploitation of Mlo powdery mildew resistance in barley. Euphytica.

[20] Ma HX, Bai GH, Gill BS, Hart LP (2006). Deletion of a chromosome arm altered wheat resistance to Fusarium head blight and deoxynivalenol accumulation in Chinese Spring. Plant Dis.

[21] Garvin DF, Porter H, Blankenheim ZJ, Chao S, Dill-Macky R (2015). A spontaneous segmental deletion from chromosome arm 3DL enhances Fusarium head blight resistance in wheat. Genome.

[22] Hales B, Steed A, Giovannelli V, Burt C, Lemmens M, Molnár-Láng M (2020). Type II Fusarium head blight susceptibility conferred by a region on wheat chromosome 4D. J Exp Bot.

[23] Chhabra B, Tiwari V, Gill BS, Dong Y, Rawat N (2021). Discovery of a susceptibility factor for Fusarium head blight on chromosome 7A of wheat. Theor Appl Genet.

[24] Nalam VJ, Alam S, Keereetaweep J, Venables B, Burdan D, Lee H (2015). Facilitation of Fusarium graminearum infection by 9-lipoxygenases in Arabidopsis and wheat. MPMI.

[25] Gordon CS, Rajagopalan N, Risseeuw EP, Surpin M, Ball FJ, Barber CJ (2016). Characterization of Triticum aestivum abscisic acid receptors and a possible role for these in mediating Fusairum head blight susceptibility in wheat. Ma W, editor. PLoS One.

[26] Hu LQ, Mu JJ, Su PS, Wu HY, Yu GH, Wang GP (2018). Multi-functional roles of TaSSI2 involved in Fusarium head blight and powdery mildew resistance and drought tolerance. J Integr Agric.

[27] Li G, Zhou J, Jia H, Gao Z, Fan M, Luo Y (2019). Mutation of a histidine-rich calcium-binding-protein gene in wheat confers resistance to Fusarium head blight. Nat Genet.

[28] Su Z, Bernardo A, Tian B, Chen H, Wang S, Ma H (2019). A deletion mutation in TaHRC confers Fhb1 resistance to Fusarium head blight in wheat. Nat Genet.

[29] Brauer EK, Balcerzak M, Rocheleau H, Leung W, Schernthaner J, Subramaniam R (2020). Genome editing of a deoxynivalenol-induced transcription factor confers resistance to Fusarium graminearum in wheat. MPMI.

[30] Su P, Zhao L, Li W, Zhao J, Yan J, Ma X (2021). Integrated metabolo-transcriptomics and functional characterization reveals that the wheat auxin receptor TIR1 negatively regulates defense against Fusarium graminearum. J Integr Plant Biol.

[31] Fabre F, Rocher F, Alouane T, Langin T, Bonhomme L (2020). Searching for FHB resistances in bread wheat: susceptibility at the crossroad. Front Plant Sci.

[32] Gorash A, Armonienė R, Kazan K (2021). Can effectoromics and loss-of-susceptibility be exploited for improving Fusarium head blight resistance in wheat?. Crop J.

[33] Baillo EH, Kimotho RN, Zhang Z, Xu P (2019). Transcription factors associated with abiotic and biotic stress tolerance and their potential for crops improvement. Genes.

[34] Tran LSP, Nishiyama R, Yamaguchi-Shinozaki K, Shinozaki K (2010). Potential utilization of NAC transcription factors to enhance abiotic stress tolerance in plants by biotechnological approach. gmcrops.

[35] Khan SA, Li MZ, Wang SM, Yin HJ (2018). Revisiting the role of plant transcription factors in the battle against abiotic stress. Int J Mol Sci.

[36] Khan M, Seto D, Subramaniam R, Desveaux D (2018). Oh, the places they’ll go! A survey of phytopathogen effectors and their host targets. Plant J.

[37] Ding L, Xu H, Yi H, Yang L, Kong Z, Zhang L (2011). Resistance to hemi-biotrophic F. graminearum infection is associated with coordinated and ordered expression of diverse defense signaling pathways. PLoS One.

[38] Gottwald S, Samans B, Lück S, Friedt W (2012). Jasmonate and ethylene dependent defence gene expression and suppression of fungal virulence factors: two essential mechanisms of Fusarium head blight resistance in wheat?. BMC Genom.

[39] Erayman M, Turktas M, Akdogan G, Gurkok T, Inal B, Ishakoglu E (2015). Transcriptome analysis of wheat inoculated with Fusarium graminearum. Front Plant Sci.

[40] Nussbaumer T, Warth B, Sharma S, Ametz C, Bueschl C, Parich A (2015). Joint transcriptomic and metabolomic analyses reveal changes in the primary metabolism and imbalances in the subgenome orchestration in the bread wheat molecular response to Fusarium graminearum. G3 (Bethesda).

[41] Ding L, Li M, Li P, Cao J (2017). Comparative proteomics analysis of young spikes of wheat in response to Fusarium graminearum infection. Acta Physiol Plant.

[42] Pan Y, Liu Z, Rocheleau H, Fauteux F, Wang Y, McCartney C (2018). Transcriptome dynamics associated with resistance and susceptibility against fusarium head blight in four wheat genotypes. BMC Genom.

[43] Wang L, Li Q, Liu Z, Surendra A, Pan Y, Li Y (2018). Integrated transcriptome and hormone profiling highlight the role of multiple phytohormone pathways in wheat resistance against fusarium head blight. Zhang A, editor. PLoS One.

[44] Brauer EK, Rocheleau H, Balcerzak M, Pan Y, Fauteux F, Liu Z (2019). Transcriptional and hormonal profiling of Fusarium graminearum-infected wheat reveals an association between auxin and susceptibility. Physiol Mol Plant Pathol.

[45] Fabre F, Vignassa M, Urbach S, Langin T, Bonhomme L (2019). Time-resolved dissection of the molecular crosstalk driving *Fusarium* head blight in wheat provides new insights into host susceptibility determinism. Plant Cell Environ.

[46] Wang B, Li X, Chen W, Kong L (2019). Isobaric tags for relative and absolute quantification-based proteomic analysis of defense responses triggered by the fungal pathogen Fusarium graminearum in wheat. J Proteomics.

[47] Fabre F, Urbach S, Roche S, Langin T, Bonhomme L (2021). Proteomics-based data integration of wheat cultivars facing Fusarium graminearum strains revealed a core-responsive pattern controlling Fusarium head blight. Front Plant Sci.

[48] Yang M, Wang X, Dong J, Zhao W, Alam T, Thomashow LS (2021). Proteomics reveals the changes that contribute to Fusarium head blight resistance in wheat. Phytopathology.

[49] Rocher F, Alouane T, Philippe G, Martin ML, Label P, Langin T (2022). Fusarium graminearum infection strategy in wheat involves a highly conserved genetic program that controls the expression of a core effectome. Int J Mol Sci.

[50] Li M, Yang Z, Chang C (2022). Susceptibility is new resistance: wheat susceptibility genes and exploitation in resistance breeding. Agriculture.

[51] Chen H, Su Z, Tian B, Hao G, Trick HN, Bai G (2022). TaHRC suppresses the calcium-mediated immune response and triggers wheat Fusarium head blight susceptibility. Plant Physiol.

[52] Low YC, Lawton MA, Di R (2022). Ethylene insensitive 2 (EIN2) as a potential target gene to enhance Fusarium head blight disease resistance. Plant Sci.

[53] Rushton PJ, Somssich IE, Ringler P, Shen QJ (2010). WRKY transcription factors. Trends Plant Sci.

[54] Ng DWK, Abeysinghe JK, Kamali M (2018). Regulating the regulators: the control of transcription factors in plant defense signaling. Int J Mol Sci.

[55] Bian Z, Gao H, Wang C (2021). NAC Transcription factors as positive or negative regulators during ongoing battle between pathogens and our food crops. Int J Mol Sci.

[56] Soni N, Altartouri B, Hegde N, Duggavathi R, Nazarian-Firouzabadi F, Kushalappa AC (2021). TaNAC032 transcription factor regulates lignin-biosynthetic genes to combat Fusarium head blight in wheat. Plant Sci.

[57] Perochon A, Kahla A, Vranić M, Jia J, Malla KB, Craze M (2019). A wheat NAC interacts with an orphan protein and enhances resistance to Fusarium head blight disease. Plant Biotechnol J.

[58] Kage U, Yogendra KN, Kushalappa AC (2017). TaWRKY70 transcription factor in wheat QTL-2DL regulates downstream metabolite biosynthetic genes to resist Fusarium graminearum infection spread within spike. Sci Rep.

[59] Zhang XM, Zhang Q, Pei CL, Li X, Huang XL, Chang CY (2018). TaNAC2 is a negative regulator in the wheat-stripe rust fungus interaction at the early stage. Physiol Mol Plant Pathol.

[60] Wang B, Wei J, Song N, Wang N, Zhao J, Kang Z (2018). A novel wheat NAC transcription factor, TaNAC30, negatively regulates resistance of wheat to stripe rust. J Integr Plant Biol.

[61] Eulgem T, Somssich IE (2007). Networks of WRKY transcription factors in defense signaling. Curr Opin Plant Biol.

[62] Nuruzzaman M, Sharoni AM, Kikuchi S (2013). Roles of NAC transcription factors in the regulation of biotic and abiotic stress responses in plants. Front Microbiol.

[63] Jiang J, Ma S, Ye N, Jiang M, Cao J, Zhang J (2017). WRKY transcription factors in plant responses to stresses. J Integr Plant Biol.

[64] Paul P, Singh SK, Patra B, Liu X, Pattanaik S, Yuan L (2020). Mutually regulated AP2/ERF gene clusters modulate biosynthesis of specialized metabolites in plants. Plant Physiol.

[65] Bemer M, van Dijk ADJ, Immink RGH, Angenent GC (2017). Cross-family transcription factor interactions: an additional layer of gene regulation. Trends Plant Sci.

[66] Cao Y, Li K, Li Y, Zhao X, Wang L (2020). MYB Transcription factors as regulators of secondary metabolism in plants. Biology.

[67] Kloppholz S, Kuhn H, Requena N (2011). A secreted fungal effector of glomus intraradices promotes symbiotic biotrophy. Curr Biol.

[68] Qin J, Wang K, Sun L, Xing H, Wang S, Li L (2018). The plant-specific transcription factors CBP60g and SARD1 are targeted by a Verticillium secretory protein VdSCP41 to modulate immunity. eLife.

[69] Tanaka S, Brefort T, Neidig N, Djamei A, Kahnt J, Vermerris W (2014). A secreted Ustilago maydis effector promotes virulence by targeting anthocyanin biosynthesis in maize. Kliebenstein DJ, editor. eLife.

[70] Paudel B, Zhuang Y, Galla A, Dahal S, Qiu Y, Ma A (2020). WFhb1-1 plays an important role in resistance against Fusarium head blight in wheat. Sci Rep.

[71] Büschges R, Hollricher K, Panstruga R, Simons G, Wolter M, Frijters A (1997). The barley Mlo gene: a novel control element of plant pathogen resistance. Cell.

[72] Engelhardt S, Stam R, Hückelhoven R (2018). Good riddance? Breaking disease susceptibility in the era of new breeding technologies. Agronomy.

[73] Garcia-Ruiz H, Szurek B, Van den Ackerveken G (2021). Stop helping pathogens: engineering plant susceptibility genes for durable resistance. Curr Opin Biotechnol.

[74] Yu G, Chen Q, Wang X, Meng X, Yu Y, Fan H (2019). Mildew resistance locus O genes CsMLO1 and CsMLO2 are negative modulators of the Cucumis sativus defense response to Corynespora cassiicola. Int J Mol Sci.

[75] Robert-Seilaniantz A, Grant M, Jones JDG (2011). Hormone crosstalk in plant disease and defense: more than just jasmonate-salicylate antagonism. Annu Rev Phytopathol.

[76] Zhang Y, Zhao L, Zhao J, Li Y, Wang J, Guo R (2017). S5H/DMR6 encodes a salicylic acid 5-hydroxylase that fine-tunes salicylic acid homeostasis. Plant Physiol.

[77] Low YC, Lawton MA, Di R (2020). Validation of barley 2OGO gene as a functional orthologue of Arabidopsis DMR6 gene in Fusarium head blight susceptibility. Sci Rep.

[78] Huai B, Yuan P, Ma X, Zhang X, Jiang L, Zheng P (2022). Sugar transporter TaSTP3 activation by TaWRKY19/61/82 enhances stripe rust susceptibility in wheat. New Phytol.

[79] Carella P, Evangelisti E, Schornack S (2018). Sticking to it: phytopathogen effector molecules may converge on evolutionarily conserved host targets in green plants. Curr Opin Plant Biol.

[80] Ceulemans E, Ibrahim HMM, De Coninck B, Goossens A (2021). Pathogen effectors: exploiting the promiscuity of plant signaling hubs. Trends Plant Sci.

[81] Fabre F, Bormann J, Urbach S, Roche S, Langin T, Bonhomme L (2019). Unbalanced roles of fungal aggressiveness and host cultivars in the establishment of the Fusarium head blight in bread wheat. Front Microbiol.

[82] GeT: Génomique & Transcriptomique. 2021. https://get.genotoul.fr/. Accessed 7 Feb 2022.

[83] CEA - Institut de biologie François Jacob: Genoscope - Centre National de Séquençage. 2021. https://www.cea.fr/drf/ifrancoisjacob/Pages/Departements/Genoscope.aspx. Accessed 7 Feb 2022.

[84] UCA: Mésocentre. 2021. https://mesocentre.uca.fr/accueil-mesocentre. Accessed 1 Sep 2021.

[85] CEA - HPC: TGCC. 2021. https://www-hpc.cea.fr/en/TGCC.html. Accessed 1 Sep 2021.

[86] Krueger F. Babraham Bioinformatics - Trim Galore. 2021. https://www.bioinformatics.babraham.ac.uk/projects/trim_galore/.

[87] Dobin A, Davis CA, Schlesinger F, Drenkow J, Zaleski C, Jha S (2013). STAR: ultrafast universal RNA-seq aligner. Bioinformatics.

[88] Pruitt KD, Tatusova T, Maglott DR (2005). NCBI Reference Sequence (RefSeq): a curated non-redundant sequence database of genomes, transcripts and proteins. Nucleic Acids Res.

[89] Kopylova E, Noé L, Touzet H (2012). SortMeRNA: fast and accurate filtering of ribosomal RNAs in metatranscriptomic data. Bioinformatics.

[90] Ensembl Plants: Triticum_aestivum - GCA_900519105.1 assembly. 2018. https://plants.ensembl.org/Triticum_aestivum/Info/Index. Accessed 31 Aug 2021.

[91] Ensembl Fungi: Fusarium_graminearum - PH1 GCA_900044135.1 assembly. 2021. http://fungi.ensembl.org/Fusarium_graminearum/Info/Index. Accessed 31 Aug 2021.

[92] IWGSC: RefSeq v1.1 Annotation of GCA_900519105.1 genome assembly. 2018. https://urgi.versailles.inra.fr/download/iwgsc/IWGSC_RefSeq_Annotations/v1.1/. Accessed 31 Aug 2021.

[93] Liao Y, Smyth GK, Shi W (2014). featureCounts: an efficient general purpose program for assigning sequence reads to genomic features. Bioinformatics.

[94] R: The R Project for statistical computing. 2022. https://www.r-project.org/. Accessed 20 Jun 2022.

[95] Robinson MD, Oshlack A (2010). A scaling normalization method for differential expression analysis of RNA-seq data. Genome Biol.

[96] Robinson MD, McCarthy DJ, Smyth GK (2010). edgeR: a Bioconductor package for differential expression analysis of digital gene expression data. Bioinformatics.

[97] Lambert I, Paysant-Le Roux C, Colella S, Martin-Magniette ML (2020). DiCoExpress: a tool to process multifactorial RNAseq experiments from quality controls to co-expression analysis through differential analysis based on contrasts inside GLM models. Plant Methods.

[98] Benjamini Y, Hochberg Y (1995). Controlling the false discovery rate: a practical and powerful approach to multiple testing. J R Stat Soc Series B Stat Methodol.

[99] Bion R. ggradar. 2022. https://github.com/ricardo-bion/ggradar. Accessed 21 Jun 2022.

[100] Love MI, Huber W, Anders S (2014). Moderated estimation of fold change and dispersion for RNA-seq data with DESeq2. Genome Biol.

[101] Kolde R. pheatmap. 2021. https://github.com/raivokolde/pheatmap. Accessed 1 Sep 2021.

[102] Murtagh F, Legendre P (2014). Ward’s hierarchical agglomerative clustering method: which algorithms implement ward’s criterion?. J Classif.

[103] Rohart F, Gautier B, Singh A, Cao KAL (2017). mixOmics: an R package for ‘omics feature selection and multiple data integration. PLoS Comput Biol.

[104] Lê S, Josse J, Husson F (2008). FactoMineR: an R package for multivariate analysis. J Stat Softw.

[105] Cassan O, Lèbre S, Martin A (2021). Inferring and analyzing gene regulatory networks from multi-factorial expression data: a complete and interactive suite. BMC Genom.

[106] Huynh-Thu VA, Irrthum A, Wehenkel L, Geurts P (2010). Inferring regulatory networks from expression data using tree-based methods. Isalan M, editor. PLoS One.

[107] Blondel VD, Guillaume JL, Lambiotte R, Lefebvre E (2008). Fast unfolding of communities in large networks. J Stat Mech.

[108] Almende BV, Thieurmel B. DataStorm: visNetwork. 2022. https://github.com/datastorm-open/visNetwork. Accessed 21 Jun 2022.

[109] Durinck S, Huber W, Davis S, Pepin F, Buffalo VS, Smith M. biomaRt: interface to BioMart databases (i.e. Ensembl). Bioconductor version: Release (3.15). 2022. https://bioconductor.org/packages/biomaRt/. Accessed 20 Jun 2022.

[110] Carlson M, Pagès H. AnnotationForge: tools for building SQLite-based annotation data packages. Bioconductor version: Release (3.15). 2022. https://bioconductor.org/packages/AnnotationForge. Accessed 20 Jun 2022.

[111] Zheng Y, Jiao C, Sun H, Rosli HG, Pombo MA, Zhang P (2016). iTAK: a program for genome-wide prediction and classification of plant transcription factors, transcriptional regulators, and protein kinases. Mol Plant.

[112] Ramírez-González RH, Borrill P, Lang D, Harrington SA, Brinton J, Venturini L (2018). The transcriptional landscape of polyploid wheat. Science.

[113] Boutet E, Lieberherr D, Tognolli M, Schneider M, Bairoch A, Edwards D (2007). UniProtKB/Swiss-Prot. Plant bioinformatics: methods in molecular biology™.

[114] Altschul SF, Gish W, Miller W, Myers EW, Lipman DJ (1990). Basic local alignment search tool. J Mol Biol.

[115] R Core Team. Hypergeometric function – Rdocumentation. 2022. https://www.rdocumentation.org/packages/stats/versions/3.6.2/topics/Hypergeometric. Accessed 4 Jun 2022.

[116] Wu T, Hu E, Xu S, Chen M, Guo P, Dai Z (2021). clusterProfiler 4.0: a universal enrichment tool for interpreting omics data. Innovation.

[117] Gel B, Serra E (2017). karyoploteR: an R/Bioconductor package to plot customizable genomes displaying arbitrary data. Bioinformatics.

[118] Gilligan CA (1990). Comparison of disease progress curves. New Phytol.

[119] Pinheiro J, Bates D, R Core Team. nlme: linear and nonlinear mixed effects models. 2022. https://cran.r-project.org/web/packages/nlme/index.html. Accessed 23 Jun 2022.

[120] Interaction between the wheat cultivar ‘Recital’ and 3 Fusarium graminearum strains. NCBI BioProject accession PRJEB59062. 2024. https://www.ncbi.nlm.nih.gov/bioproject/PRJEB59062.

[121] Responses of different wheat cultivars to Fusarium Head Blight (RNA-Sequencing). NCBI BioProject accession. 2024. https://www.ncbi.nlm.nih.gov/bioproject/PRJEB59238.

