# Supplementary material for: Integrative systems biology of wheat susceptibility to Fusarium graminearum uncovers a conserved gene regulatory network and identifies master regulators targeted by fungal core effectors
Source: BMC Biol. 2024 Mar 5;22:53. doi: 10.1186/s12915-024-01852-x (PMC10916188; doi:10.1186/s12915-024-01852-x)
Supplement: Supplementary file 1 — Additional file 1: Table S1. Resume table of the statistical analysis performed on 'Recital' genes when facing one of the three different F. graminearum strains. Table S2. Expression patterns for each identified TF family that were FHB-responsive in 'Recital' facing one of the three F. graminearum strains. Table S3. A GO BP term enrichment results on the gene set under-expressed in FHB samples compared with controls. B GO BP term enrichment results on the gene set over-expressed in FHB samples compared with controls. Table S4. Resume Table of the master regulators identified in the TF regulatory network of the FHB responses. Table S5. Resume table of the identified known S genes. [file 12915_2024_1852_MOESM1_ESM.pdf]

Figure S1

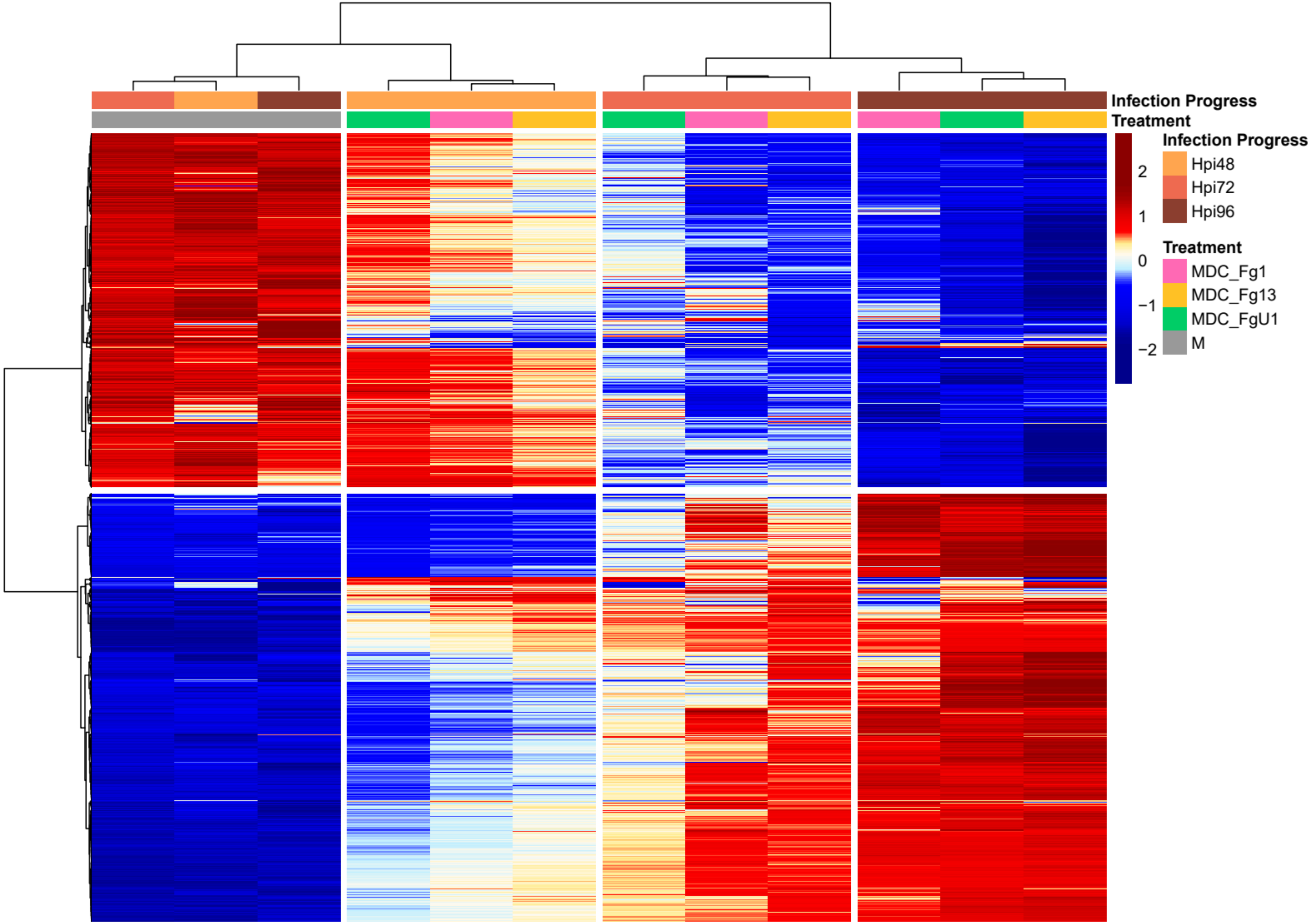

Infection Progress
Treatment
Infection Progress
Hpi48
Hpi72
Hpi96
Treatment
MDC_Fg1
MDC_Fg13
MDC_FgU1
M
2
1
0
−1
−2

Figure S1. Expression regulation patterns of the All strain -responsive gene set in 'Recital' along the time course for control and infected samples. The structure of gene and sample data sets were determined by HAC based on Ward's minimum variance method using the z-score transformed gene expression values. Heatmap color scales represent the z-score transformed expression values of the genes from the All strain -responsive gene set (26,779 genes) for each sample. The clustering on top of the heatmap represents the experimental conditions which are labeled according to the factors Time Course (48hpi, 72hpi, 96hpi) and Treatment (MDC_Fg1, MDC_Fg13, MDC_FgU1, Mock). The clustering on the right side of the heatmap represents the clustering of genes.

Figure S2

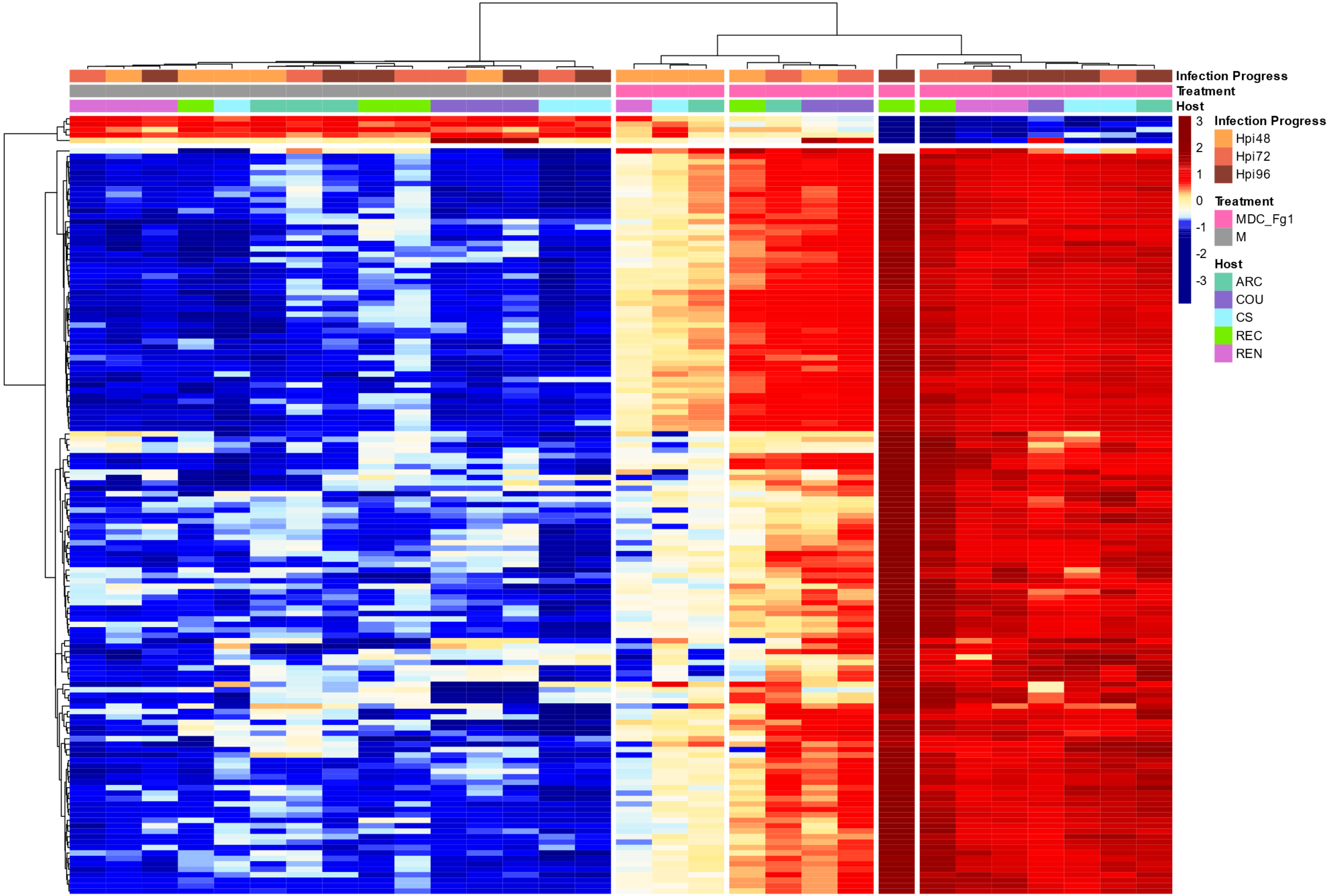

Infection Progress
Treatment
Host
3
2
1
0
-1
-2
-3
Infection Progress
Hpi48
Hpi72
Hpi96
Treatment
MDC_Fg1
M
Host
ARC
COU
CS
REC
REN

Figure S2. Expression regulation patterns of the 142 susceptibility genes targeted by *F. graminearum* nuclear effectors that are differentially expressed in response to FHB in the 5 wheat cultivars (HostV). The structure of gene and sample data sets were determined by HAC based on Ward’s minimum variance method using the z-score transformed gene expression values. Heatmap color scales represent the z-score transformed expression values of the putative susceptibility genes for each sample. The clustering on top of the heatmap represents the experimental conditions which are labeled according to the factors Infection Progress, Treatment and Cultivar.

Figure S3A

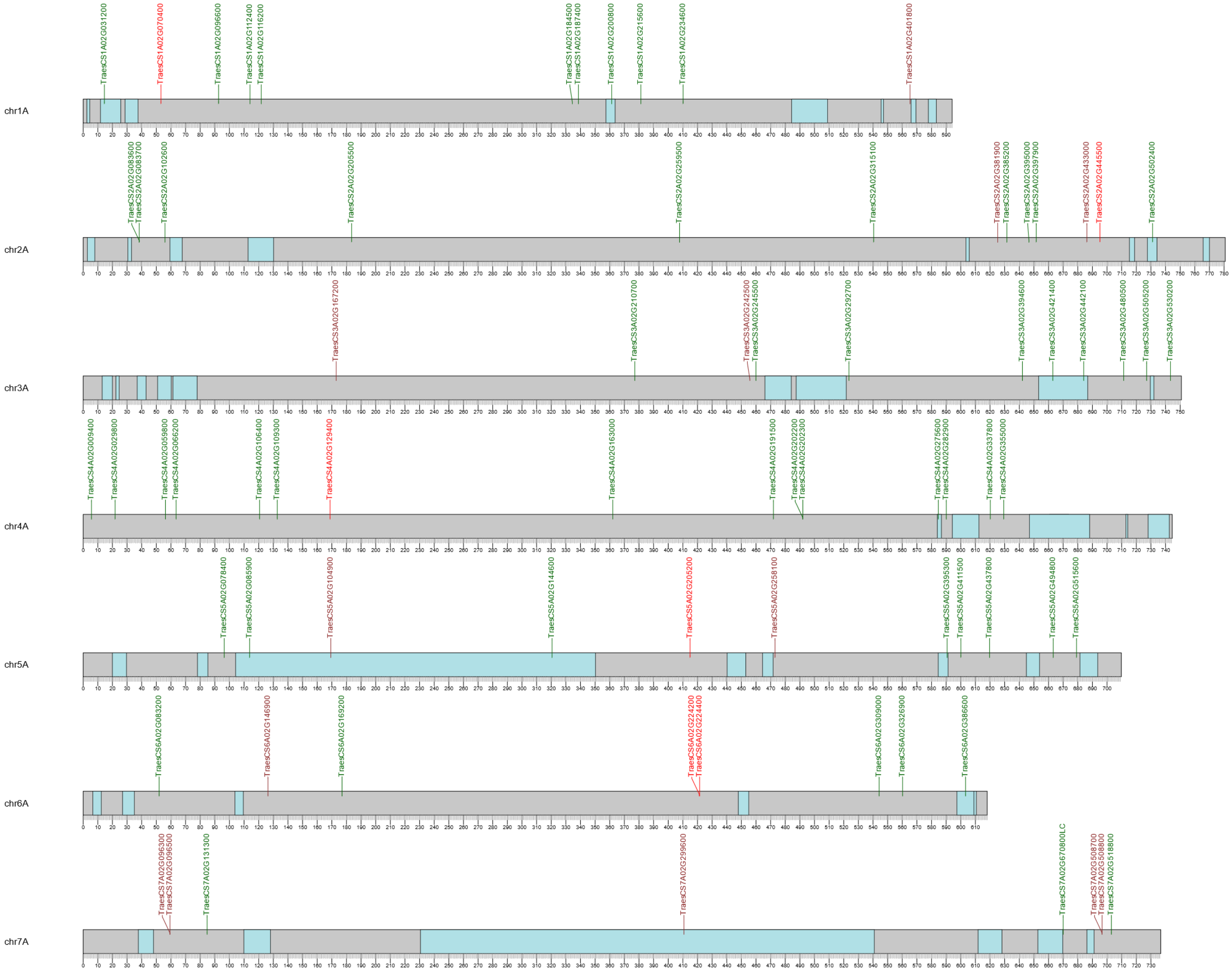

chr1A
TraesCS1A02G031200
TraesCS1A02G070400
TraesCS1A02G096600
TraesCS1A02G112400
TraesCS1A02G116200
TraesCS1A02G184500
TraesCS1A02G187400
TraesCS1A02G200800
TraesCS1A02G215600
TraesCS1A02G234600
TraesCS1A02G401800
chr2A
TraesCS2A02G083600
TraesCS2A02G083700
TraesCS2A02G102600
TraesCS2A02G205500
TraesCS2A02G259500
TraesCS2A02G315100
TraesCS2A02G381900
TraesCS2A02G385200
TraesCS2A02G395000
TraesCS2A02G397900
TraesCS2A02G433000
TraesCS2A02G445500
TraesCS2A02G502400
chr3A
TraesCS3A02G167200
TraesCS3A02G210700
TraesCS3A02G242500
TraesCS3A02G245500
TraesCS3A02G292700
TraesCS3A02G394600
TraesCS3A02G421400
TraesCS3A02G442100
TraesCS3A02G480500
TraesCS3A02G505200
TraesCS3A02G530200
chr4A
TraesCS4A02G009400
TraesCS4A02G029800
TraesCS4A02G059800
TraesCS4A02G066200
TraesCS4A02G106400
TraesCS4A02G109300
TraesCS4A02G129400
TraesCS4A02G163000
TraesCS4A02G191500
TraesCS4A02G202200
TraesCS4A02G202300
TraesCS4A02G275600
TraesCS4A02G282900
TraesCS4A02G337800
TraesCS4A02G355000
chr5A
TraesCS5A02G078400
TraesCS5A02G085900
TraesCS5A02G104900
TraesCS5A02G144600
TraesCS5A02G205200
TraesCS5A02G258100
TraesCS5A02G395300
TraesCS5A02G411500
TraesCS5A02G437800
TraesCS5A02G494800
TraesCS5A02G515600
chr6A
TraesCS6A02G083200
TraesCS6A02G146900
TraesCS6A02G169200
TraesCS6A02G224200
TraesCS6A02G224400
TraesCS6A02G309000
TraesCS6A02G326900
TraesCS6A02G386600
chr7A
TraesCS7A02G096300
TraesCS7A02G096500
TraesCS7A02G131300
TraesCS7A02G299600
TraesCS7A02G670800LC
TraesCS7A02G508700
TraesCS7A02G508800
TraesCS7A02G518800

Figure S3B

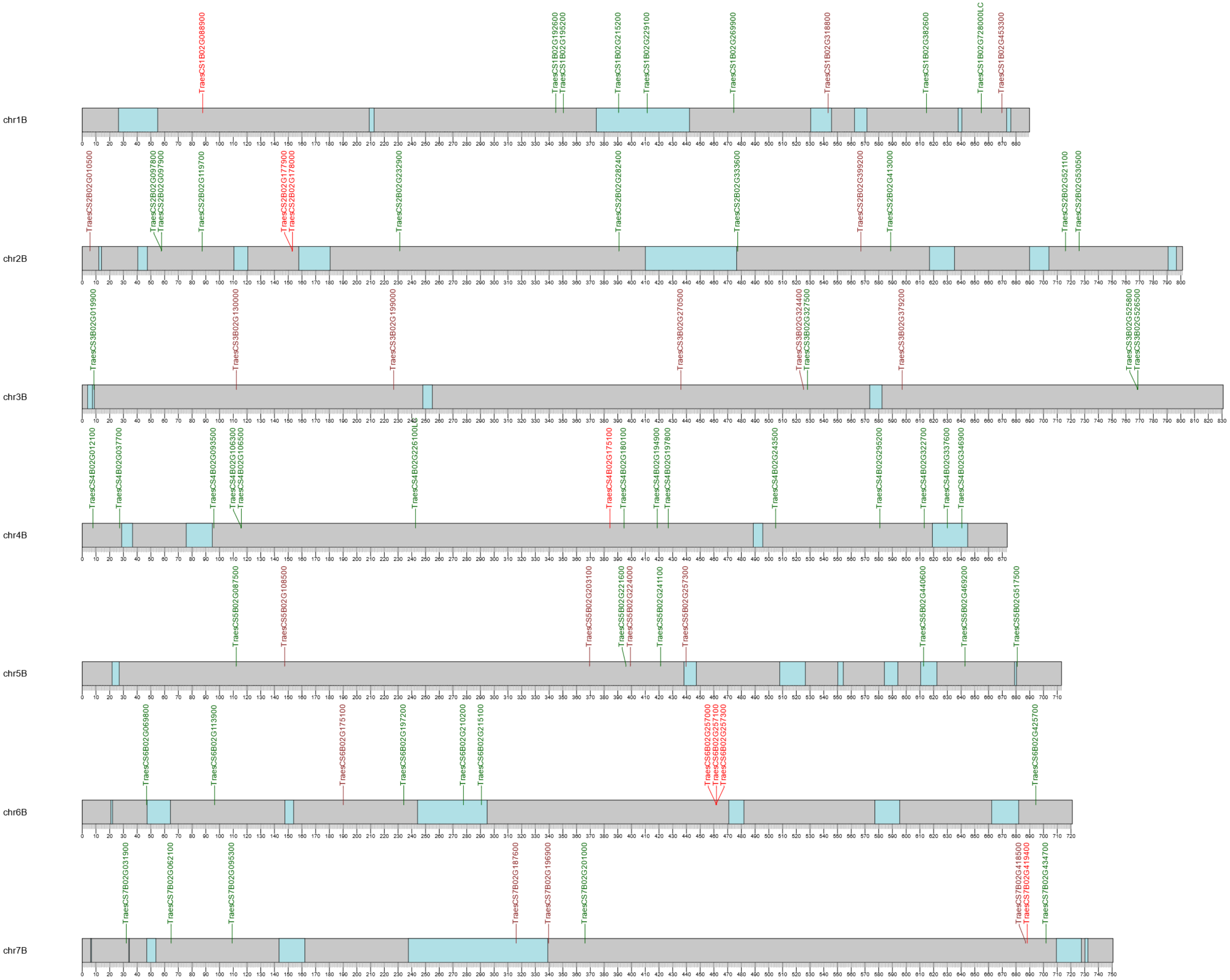
chr1B
TraesCS1B02G088900
TraesCS1B02G192600
TraesCS1B02G195200
TraesCS1B02G215200
TraesCS1B02G229100
TraesCS1B02G269900
TraesCS1B02G318800
TraesCS1B02G382600
TraesCS1B02G728000LC
TraesCS1B02G453300
chr2B
TraesCS2B02G010500
TraesCS2B02G097800
TraesCS2B02G097900
TraesCS2B02G119700
TraesCS2B02G177900
TraesCS2B02G178000
TraesCS2B02G232900
TraesCS2B02G282400
TraesCS2B02G333600
TraesCS2B02G399200
TraesCS2B02G413000
TraesCS2B02G521100
TraesCS2B02G530500
chr3B
TraesCS3B02G019900
TraesCS3B02G130000
TraesCS3B02G199000
TraesCS3B02G270500
TraesCS3B02G324400
TraesCS3B02G327500
TraesCS3B02G379200
TraesCS3B02G525800
TraesCS3B02G526500
chr4B
TraesCS4B02G012100
TraesCS4B02G037700
TraesCS4B02G093500
TraesCS4B02G106300
TraesCS4B02G106500
TraesCS4B02G226100
TraesCS4B02G175100
TraesCS4B02G180100
TraesCS4B02G194900
TraesCS4B02G197800
TraesCS4B02G243500
TraesCS4B02G295200
TraesCS4B02G322700
TraesCS4B02G337600
TraesCS4B02G346900
chr5B
TraesCS5B02G087500
TraesCS5B02G108500
TraesCS5B02G203100
TraesCS5B02G221600
TraesCS5B02G224000
TraesCS5B02G241100
TraesCS5B02G257300
TraesCS5B02G440600
TraesCS5B02G469200
TraesCS5B02G517500
chr6B
TraesCS6B02G069800
TraesCS6B02G113900
TraesCS6B02G175100
TraesCS6B02G197200
TraesCS6B02G210200
TraesCS6B02G215100
TraesCS6B02G257000
TraesCS6B02G257100
TraesCS6B02G257300
TraesCS6B02G425700
chr7B
TraesCS7B02G031900
TraesCS7B02G062100
TraesCS7B02G095300
TraesCS7B02G187600
TraesCS7B02G196900
TraesCS7B02G201000
TraesCS7B02G418500
TraesCS7B02G419400
TraesCS7B02G434700

Figure S3C

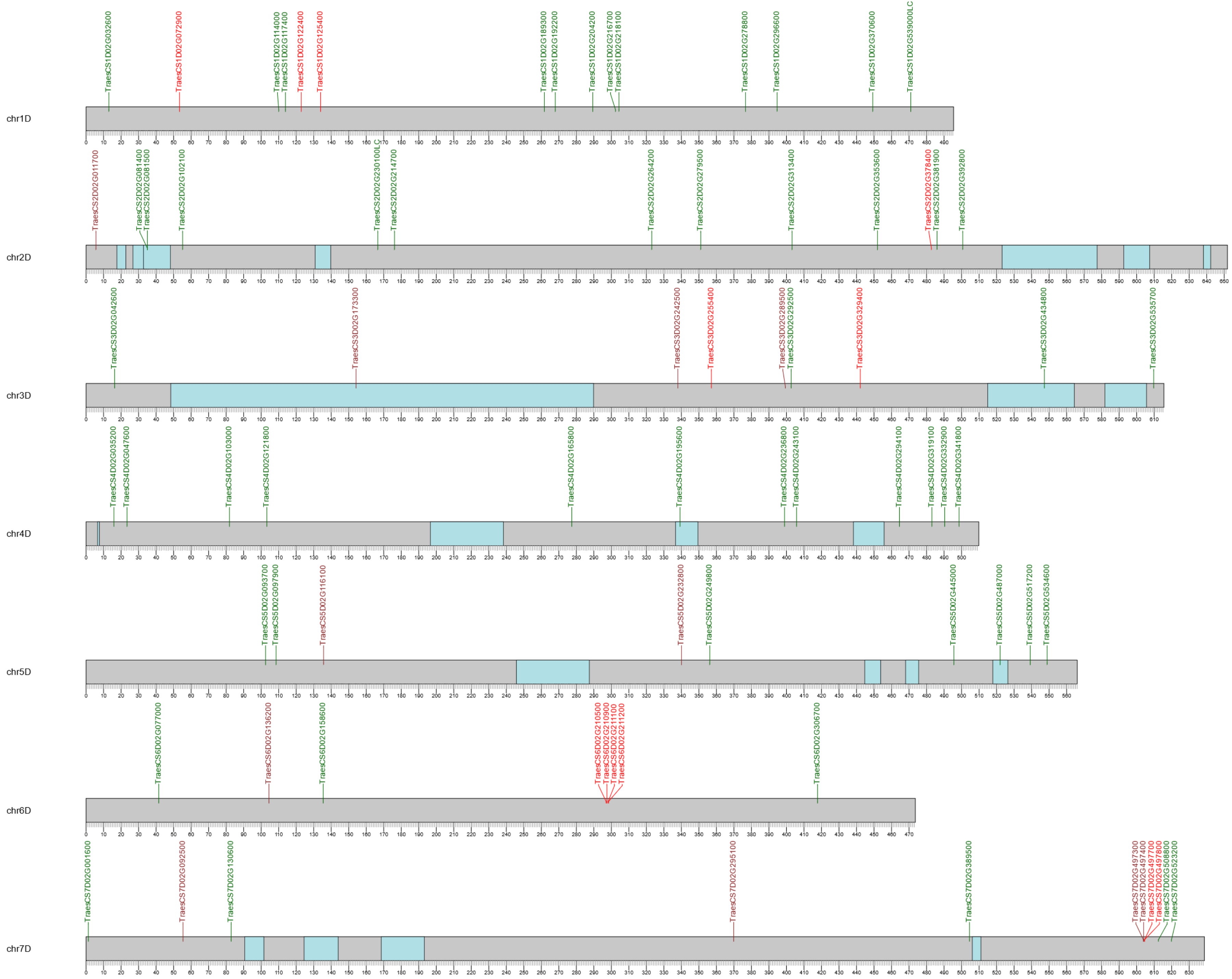

chr1D
TraesCS1D02G032600
TraesCS1D02G072900
TraesCS1D02G114000
TraesCS1D02G117400
TraesCS1D02G122400
TraesCS1D02G125400
TraesCS1D02G189300
TraesCS1D02G192200
TraesCS1D02G204200
TraesCS1D02G216700
TraesCS1D02G218100
TraesCS1D02G278800
TraesCS1D02G296600
TraesCS1D02G370600
TraesCS1D02G539000LC
chr2D
TraesCS2D02G011700
TraesCS2D02G081400
TraesCS2D02G081500
TraesCS2D02G102100
TraesCS2D02G230100LC
TraesCS2D02G214700
TraesCS2D02G264200
TraesCS2D02G279500
TraesCS2D02G313400
TraesCS2D02G353600
TraesCS2D02G378400
TraesCS2D02G381900
TraesCS2D02G392800
chr3D
TraesCS3D02G042600
TraesCS3D02G173300
TraesCS3D02G242500
TraesCS3D02G255400
TraesCS3D02G289500
TraesCS3D02G292500
TraesCS3D02G329400
TraesCS3D02G434800
TraesCS3D02G535700
chr4D
TraesCS4D02G035200
TraesCS4D02G047600
TraesCS4D02G103000
TraesCS4D02G121800
TraesCS4D02G165800
TraesCS4D02G195600
TraesCS4D02G236800
TraesCS4D02G243100
TraesCS4D02G294100
TraesCS4D02G319100
TraesCS4D02G332900
TraesCS4D02G341800
chr5D
TraesCS5D02G093700
TraesCS5D02G097900
TraesCS5D02G116100
TraesCS5D02G232800
TraesCS5D02G249800
TraesCS5D02G445000
TraesCS5D02G487000
TraesCS5D02G517200
TraesCS5D02G534600
chr6D
TraesCS6D02G077000
TraesCS6D02G136200
TraesCS6D02G158600
TraesCS6D02G210500
TraesCS6D02G210900
TraesCS6D02G211100
TraesCS6D02G211200
TraesCS6D02G306700
chr7D
TraesCS7D02G001600
TraesCS7D02G092500
TraesCS7D02G130600
TraesCS7D02G295100
TraesCS7D02G389500
TraesCS7D02G497300
TraesCS7D02G497400
TraesCS7D02G497700
TraesCS7D02G497800
TraesCS7D02G508800
TraesCS7D02G523200

Figure S3. Genomic distribution of the 233 putative S genes on wheat subgenomes A (S3A), B (S3B) and D (S3C). S genes were colored according to their categories : Master TF gene (brick red), TF gene (orange), not TF gene (green). The FHB meta-QTLs built by Zheng et al., 2021 were represented by sky blue boxes directly on the chromosomes.
